# Pan-kinome of *Legionella* expanded by a bioinformatics survey

**DOI:** 10.1038/s41598-022-26109-x

**Published:** 2022-12-16

**Authors:** Marianna Krysińska, Bartosz Baranowski, Bartłomiej Deszcz, Krzysztof Pawłowski, Marcin Gradowski

**Affiliations:** 1 Department of Biochemistry and Microbiology, Warsaw University of Life Sciences — SGGW, Warsaw, Poland; 2 Laboratory of Plant Pathogenesis, Institute of Biochemistry and Biophysics, Polish Academy of Sciences, Warsaw, Poland; 3 Department of Molecular Biology, University of Texas Southwestern Medical Center, Dallas, TX USA; 4 Department of Translational Medicine, Lund University, Lund, Sweden; 5 Howard Hughes Medical Institute, Dallas, TX, USA

**Keywords:** Kinases, Biochemistry, Microbiology, Pathogens, Protein function predictions, Protein structure predictions, Computational biology and bioinformatics, Protein sequence analyses

## Abstract

The pathogenic *Legionella* bacteria are notorious for delivering numerous effector proteins into the host cell with the aim of disturbing and hijacking cellular processes for their benefit. Despite intensive studies, many effectors remain uncharacterized. Motivated by the richness of *Legionella* effector repertoires and their oftentimes atypical biochemistry, also by several known atypical *Legionella* effector kinases and pseudokinases discovered recently, we undertook an in silico survey and exploration of the pan-kinome of the *Legionella* genus, i.e., the union of the kinomes of individual species. In this study, we discovered 13 novel (pseudo)kinase families (all are potential effectors) with the use of non-standard bioinformatic approaches. Together with 16 known families, we present a catalog of effector and non-effector protein kinase-like families within *Legionella*, available at http://bioinfo.sggw.edu.pl/kintaro/. We analyze and discuss the likely functional roles of the novel predicted kinases. Notably, some of the kinase families are also present in other bacterial taxa, including other pathogens, often phylogenetically very distant from *Legionella*. This work highlights Nature’s ingeniousness in the pathogen–host arms race and offers a useful resource for the study of infection mechanisms.

## Introduction

The *Legionella* genus includes close to 70 species of mostly pathogenic Gram-negative bacteria^1,2^. The *Legionella* strains use several secretion systems to translocate effectors into the host cell^3,4^. Thus, these bacteria can modulate host cell signaling and metabolic processes to establish a favorable replicating environment within the host cell known as the *Legionella* Containing Vacuole (LCV). The best-known species of this genus is the human pathogen *Legionella pneumophila.* It is responsible for 80–90% of infection cases caused by all the *Legionella* species^4^. *L. pneumophila* and other *Legionella* species use up to 330 effectors^5^. Usually, *Legionella* bacteria live in natural water reservoirs although some of them are isolated from non-aquatic habitats^1,4^. In water, the bacterium infects a wide range of free-living amoeba which are the natural hosts. It can also survive in the artificial environment of human-made water systems. For *L. pneumophila*, the most frequent path of transmission to humans is through inhalation or microaspiration of water contaminated with the bacteria*.* Thus, the bacterium can reach human lungs and infect alveolar macrophages. This results in diseases such as lethal, nonspecific pneumonia (called Legionnaires’ disease) or milder flu-like Pontiac fever^4,6^. Out of the at least 69 known *Legionella* species, about 25 are associated with human infections^1^.

The *Legionella* effector proteins can affect diverse cellular processes such as cell cytoskeleton rearrangement, cell adhesion, signaling, transcription, apoptosis or metabolic processes^7^. Although a large proportion of these effectors are functionally uncharacterized, many were shown to be enzymes, e.g., kinases, proteases, phosphatases^7,8^.

Many effectors do not act individually, rather, they functionally interact once inside the host cell. For instance, the SidM effector covalently adds an adenosine monophosphate (AMP) moiety to human Rab1 protein. Next, AMP can be removed by the SidD effector, thus antagonizing the SidM effect. Many such pairs of effectors, termed metaeffectors, have been described^9^.

As protein kinases are among the basic enzymes that regulate most of the cellular processes, bacteria developed effector kinases which manipulate many processes in the cell^7,9^. Here, we focus on the Protein kinase-like superfamily (Pfam clan: CL0016) which combines protein families that share a common structure—Protein Kinase-Like fold (PKL)^10,11^. For example, *E. coli* NleH1/2 and *Salmonella* OspG effector kinases modulate the human host immune response by inhibition of the host NF-κB pathway^7^. Also, it was recently discovered that they target the microvillus protein Eps8 responsible for actin bundling. This causes a change in the structure of enterocytes and leads to diarrhea in children^12^. The recently discovered HopBF1 kinase from the plant pathogen *Pseudomonas syringae* is recognized by host HSP90 as a client. HSP90 is then phosphorylated by HopBF1 to completely inhibit the chaperone’s ATPase activity. This dampens the plant’s immune response^13^.

*Legionella* has a considerable repertoire of characterized effector kinases, including eukaryotic-like protein kinases LegK1^7^, LegK2^14^, LegK3^7^, LegK4^15^, LegK7^16^ as well as phosphatidylinositol (PI) kinases—LepB^17^, AnkK^18^ and MavQ^19^.

LegK1 is considered to work similarly to NleH/OspG, by affecting the host NF-κB pathway. Thus, LegK1 activates the noncanonical NF‐κB pathway through phosphorylation of NF-kappa-B p100 subunit, which prevents host cell apoptosis^7^. LegK2 targets the ARP2/3 complex to inhibit actin polymerization on the phagosome, thereby blocking phagosome/endosome fusion and helping to remodel phagosome into LCV^14^. LegK4 phosphorylates host Hsp70 to reduce the chaperone’s ability to refold proteins which causes inhibition of cellular protein translation^15^. LegK7 functionally mimics host Hippo kinase by activating the MOB1A protein which supports bacterial growth^16^. MavQ, LepB and AnkK are kinases that phosphorylate Phosphatidylinositol (PI) or its various derivatives on the LCV, thus assuring its proper PI-based “decoration” and contributing to the evasion of the host cell degradation pathway^17–19^. Thus, *Legionella* uses a wide range of PKL proteins that hijack host signaling and metabolic pathways, which facilitates bacterial infection.

Besides effector kinases, *Legionella* has a large set of non-effector kinases (see Suppl. Table S1), including the ancient ADCK–UbiB2–ABC1 family (lpg2905 in *L. pneumophila*) involved in synthesis of ubiquinone (cofactor Q) in bacteria^20^. The well-known HipA kinase (lpg1934 in *L. pneumophila*, see also “Results and discussion” section) promotes multidrug tolerance by blocking translation, inhibition of growth, and induction of persistence^21^. Other kinases of small molecules phosphorylate antibiotics to block their actions^21,22^*.*

Motivated by the richness of *Legionella* effector repertoires and their oftentimes atypical biochemistry, also by several atypical *Legionella* effector kinases and pseudokinases discovered by us and by others (MavQ^19^, lpg2603^23^, SidJ^24^, AnkK^18^, LepB^17^), we undertook an in silico survey and exploration of the pan-kinome of the *Legionella* genus.

**Figure 1:**
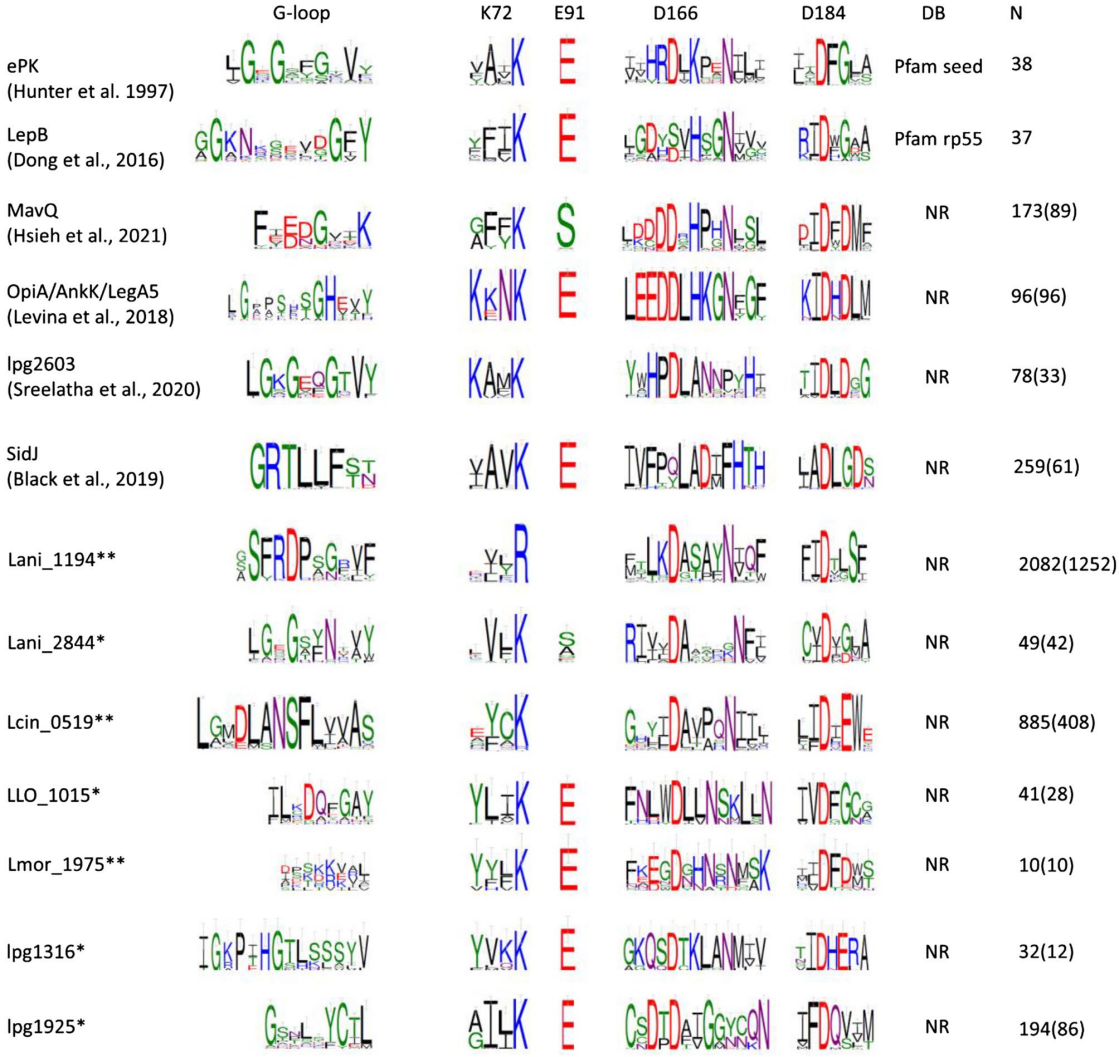
Most *Legionella* kinase-like families have conserved active site motifs. Sequence logos of active site motifs for selected families. Also, the “classic” kinases (ePK) shown. Residue numbering (top row) according to standard protein kinase A (PKA) nomenclature^26^. Asterisks denote the novel *Legionella* kinases. Double asterisks denote the novel families discussed in detail. DB: source database of kinase sequences used for the logos^27^. N indicates numbers of homologous sequences from BLAST search (E = 1e−4 threshold)^28–30^. In brackets—numbers of homologous sequences after CD-HIT clustering at the level of 99% sequence identity^31^ (see “Materials and methods” section). For some families, it was not possible to identify the residue corresponding to E91 of PKA.

In this study, we discovered 13 novel families (all are potential effector kinases; see Suppl. Table S4) with the use of non-standard bioinformatic approaches (Fig. 1, Suppl. Fig. S8). Together with 16 known families (representing 99 *Legionella* orthologous groups—LOGs^25^), we present a catalog of effector and non-effector *Legionella* PKL families, available at http://bioinfo.sggw.edu.pl/kintaro/. For the novel families, we focus on predicting their function, establish evolutionary history, and occurrence across the bacterial world.

## Results and discussion

### Charting kinases in the *Legionella* pan-proteome

The survey started from the *Legionella* pan-proteome with 16,416 orthologous groups of proteins from 41 species^25^. After clustering at 90% and 50% sequence identity thresholds^31^ and splitting them into fragments^32^ (see “Materials and methods” section), 21,616 sequences were analyzed by FFAS algorithm for distant similarity to kinases (Suppl. Table S2)^33^. Among the FFAS hits, 16 known protein kinase-like families were recognized by RPS-BLAST^34,35^ and from the literature (Suppl. Table S1). Thirteen FFAS kinase-like hits were not automatically recognized as such and were validated by other remote sequence similarity search methods (HHpred/HHsearch) (Suppl. Table S2)^36^, Phyre2 (Suppl. Table S2)^37^, analysis of sequence logos^27^ with secondary structure (Suppl. Fig. S8)^38^ and de novo structure modeling using the RoseTTAFold^39^ and AlphaFold2^40^ methods supplemented with structural comparisons (FATCAT^41^ and Dali^42^ servers) (Suppl. Tables S3, S9). For most of the modeled structures of the novel kinases, significant similarity to known protein kinase structures was found (see prediction summary in Suppl. Table S3). For Lmor_1975, LLO_2159, and Lsai_0337, the similarity of structure models to known kinases was partial or weak (Suppl. Table S3).

**Figure 2:**
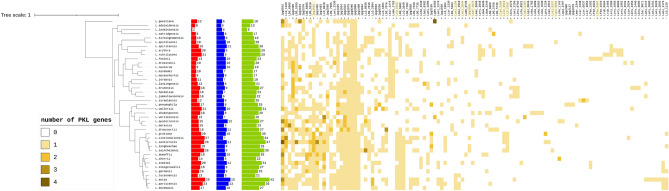
Distribution of 112 kinase LOGs among 41 *Legionella* species. Histograms on the left: counts of effector kinases shown in red, non-effectors in blue; percentage of effector kinases in a kinome shown in green. Clustered heatmap depicts the numbers of each LOG representative per species (range 0–4). Gene labels marked with plus (+) indicate effector families/LOGs. Novel families are marked by highlighted gene labels. Phylogenetic tree of *Legionella* species adapted from the publication by Burstein et al.^25,43^.

The *Legionella* species differ greatly in numbers of kinase-like families, ranging from 8 to 43. This kind of diversity among effector and non-effector repertoires is believed to result from the adaptations to infecting different hosts (e.g., different amoeba species)^25,43^. For every species, effectors form the majority of the kinome (Fig. 2).

**Figure 3:**
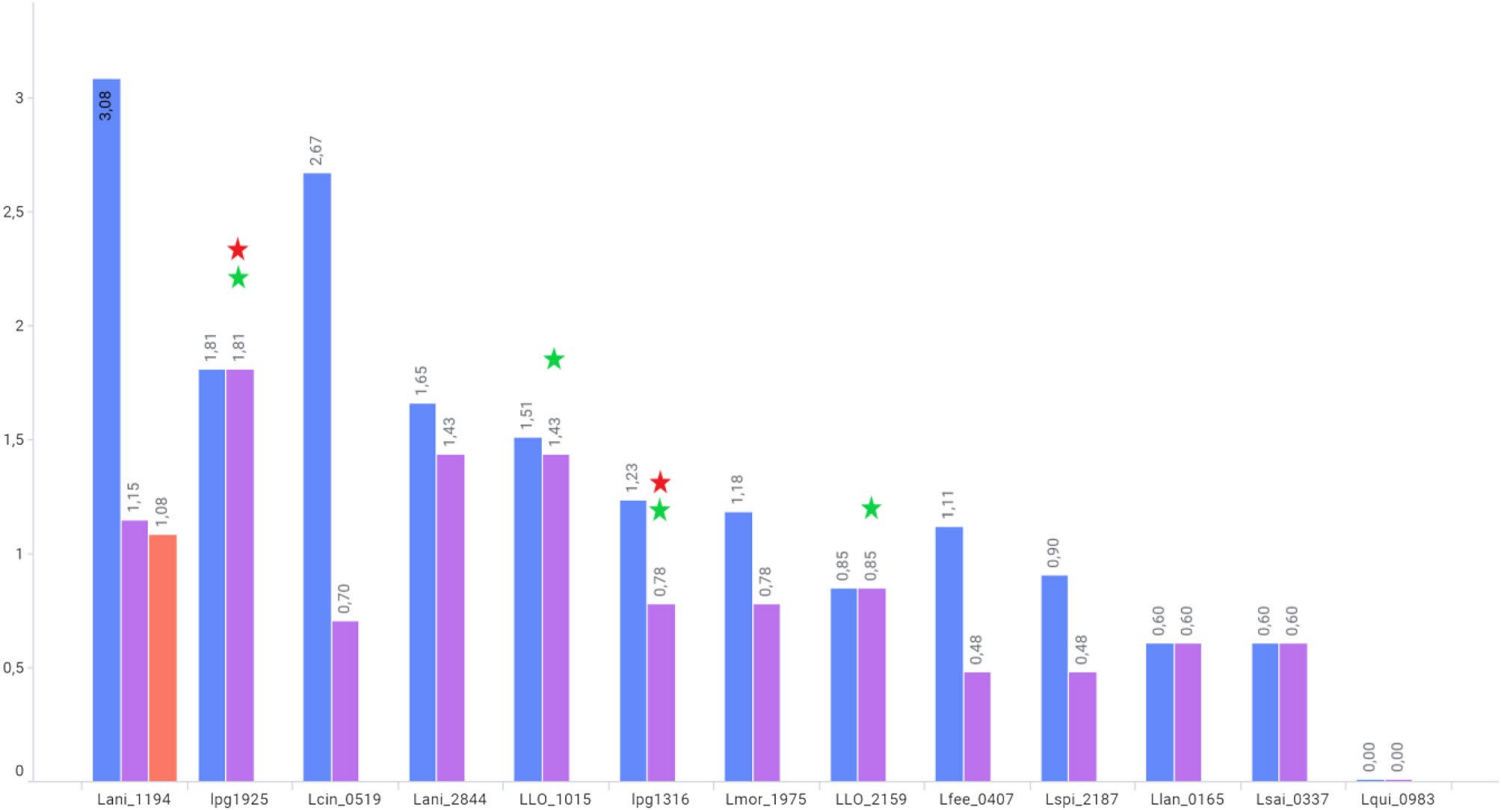
Numbers of species with homologs of novel *Legionella* kinase families. Numbers of species with homologs shown in logarithmic scale. The sequences were collected by BLAST search in the NR database (BLAST at E = 1e−4)^28–30^. Blue columns — *Bacteria*, purple — *Legionellaceae,* red — *Archaea*. Red asterisk — homologs present in *Legionella pneumophila subsp. pneumophila* str. Philadelphia 1, green asterisk — homologs in *Legionella longbeachae* NSW150.

Interestingly, some of the novel families have many hundreds of homologs outside the *Legionellaceae* family, while some are restricted to *Legionellaceae* or even a subset thereof (Fig. 3). The two families with largest numbers of homologs (Lani_1194 and Lcin_0519) are discussed in detail in a later section. Among the 112 kinase families, there are predicted effectors and non-effector kinase families (Fig. 2, Suppl. Table S4)^44–46^. In almost every species analyzed, effector kinases constitute the majority of kinome, e.g., 17 out of 25 in *L. pneumophila*.

Almost all novel families have well-preserved key kinase residues (see Fig. 1, Suppl. Fig. S8). Only in Lani_1194 the catalytic lysine K72 (PKA) is replaced by R. In Lcin_0519 and Lani_1194, the equivalent of E91 cannot be identified by sequence and structure analysis. Only in two pseudokinase cases, Llan_0165 and Lspi_2187, the catalytic aspartate D166 (PKA) is not conserved, while N171 and D184 (PKA) are conserved in all novel families.

### Sequence similarity analysis of novel kinase families

**Figure 4:**
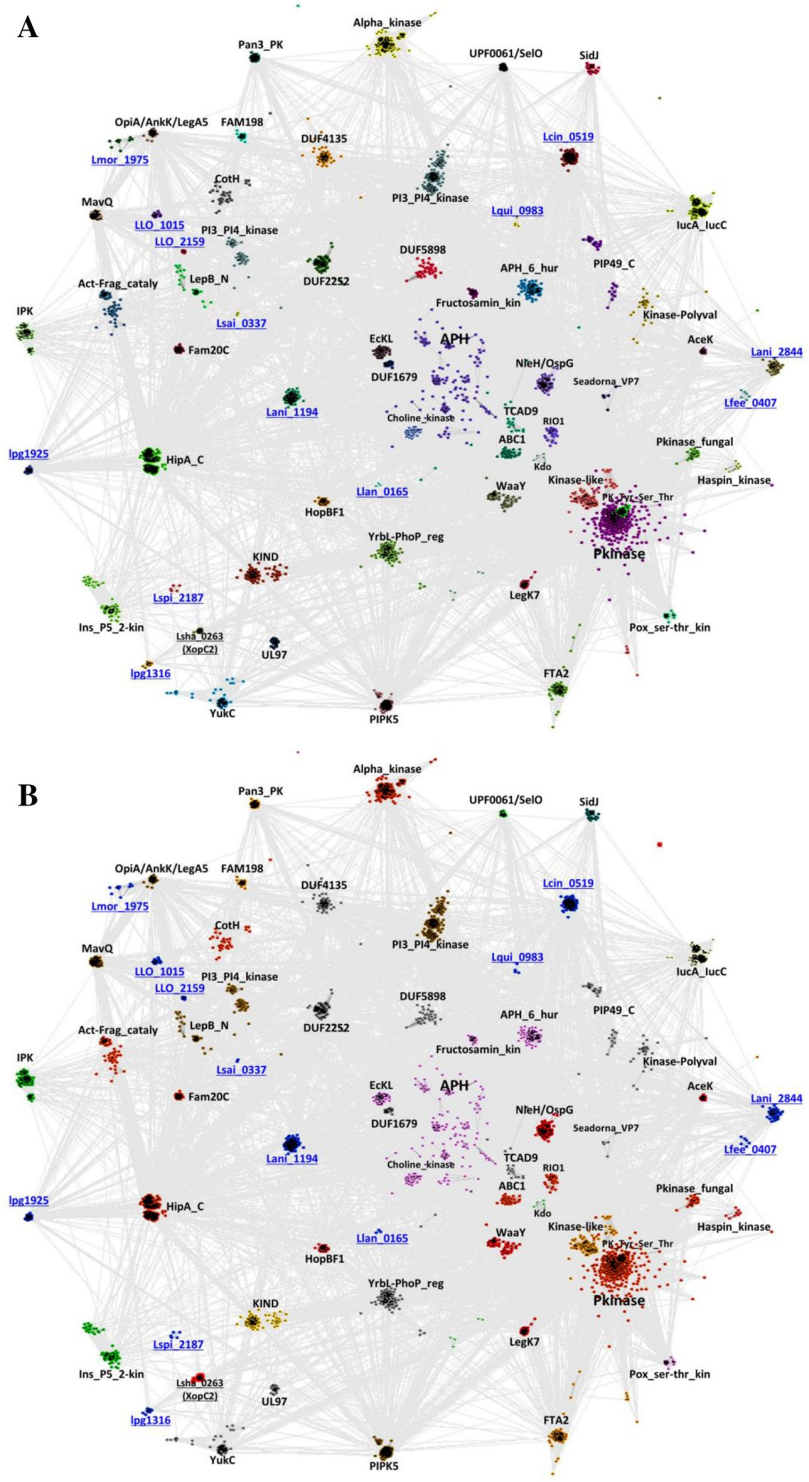
Sequence similarity graph for novel *Legionella* kinases and known PKL families from all domains of life. The CLANS graph includes representatives of all known PKL families (see “Methods” section). Graph edges represent protein sequence similarities detected by all-to-all BLAST comparisons up to the E-value of 1^47^. Pfam identifiers of selected families shown, for novel families, symbols of representative genes used. Novel families of *Legionella* (pseudo)kinases marked in blue underline. (**A**) Coloring by families. (**B**) Coloring by dominant function: red — protein phosphorylation, cyan — phospholipid phosphorylation, lime — lipopolysaccharide phosphorylation, pink — fructosamine phosphorylation, dark green — phosphorylation of inositol and derivatives, magenta — small molecule phosphorylation, brown — phosphorylation of phosphatidylinositol and derivatives, orange — pseudokinase (likely non-enzymatic functions), pale yellow — biosynthesis of small molecules, green-grey — glutamylation, light green — AMPylation (adenylylation), grey — unknown function or function predicted but unverified, blue — novel *Legionella* families.

The CLANS graph analysis (Fig. 4) allows the investigation of sequence similarity relationships between the 13 novel *Legionella* kinase families and 49 known kinase families from all the domains of life (see “Materials and methods” section)^47^. This graph may indicate distant relationships between families, which are important for understanding their evolution and functionality. The CLANS graph represents quasi-distances between sequences, based on multiple pairwise alignments built by all-to-all the BLAST sequence comparisons. This approach is used consciously, because classical phylogenetics analysis would require an unambiguous multiple sequence alignment. Achieving such an alignment of diverse and very distant families is difficult due to the presence of family-specific regions. Even structure-based alignments suffer from this problem in diverse superfamilies. The analysis shows that the novel families generally do not cluster by connectivity and proximity with established, well-studied ones. An exception are four novel families clustering with the FAM20/CotH group (Lmor_1975; LLO_1015; LLO_2159; Lsai _0337), together with known *Legionella* kinases LepB and AnkK, which suggests they may be phosphorylating derivatives of phosphatidylinositol or other lipids. However, most novel families do not cluster with PKL families of known functions, e.g., protein kinases and lipid kinases. Phylogenetic trees built for three Protein Kinase-Like groups from *Legionella* and their selected eukaryotic counterparts using structure-based sequence alignments, do support the bacterial origin of the HipA-like *Legionella* kinases, and the phosphatidylinositol kinase-like proteins while supporting the likely eukaryotic origin of the known LegK1–4 kinases (see Suppl. Fig. S15).

### Sequence similarity network suggests possible horizontal gene transfer events

**Figure 5:**
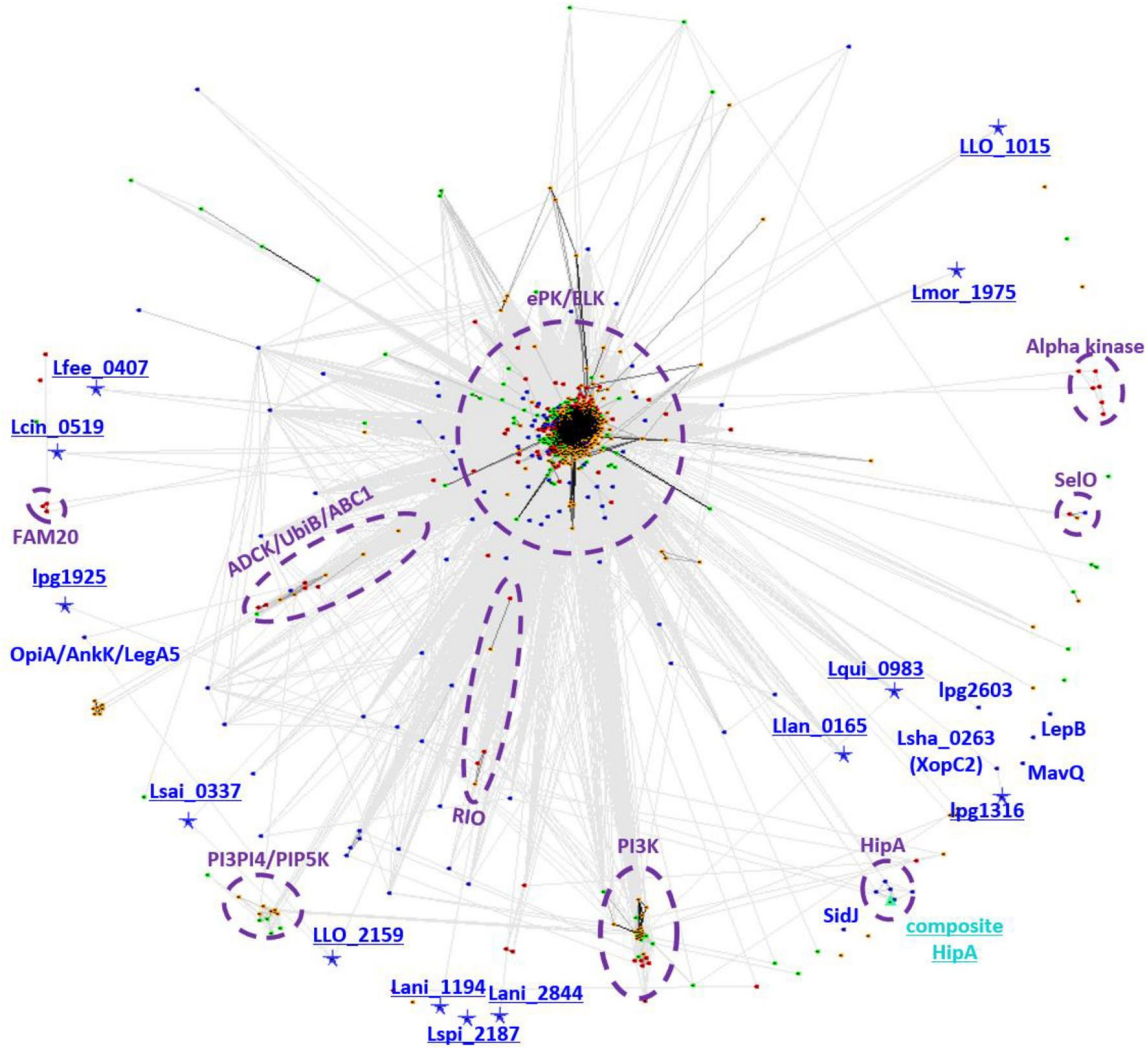
Sequence similarities: kinase-like proteins from *Legionella,* human and amoeba. The CLANS graph built as in Fig. 4 (up to BLAST E-value of 1^47^. Protein kinase-like proteins from the genus *Legionella* (blue), human (red) and amoebas — *Dictyostelium discoideum* (green) and *Acanthamoeba castellanii* (yellow). The novel kinase families are underlined and marked by asterisks. Ellipses mark selected protein families.

A CLANS sequence similarity network including all kinases from 41 *Legionella* species and full kinomes of the hosts: human and amoebas *Dictyostelium discoideum* and *Acanthamoeba castellanii* can be used for a tentative overview of evolutionary relationships (Fig. 5, Suppl. 11)^47^. The CLANS graph should be treated as an inaccurate representation of the relationship network, where the complex multidimensional network of similarities is captured on a two-dimensional graph where similar protein kinases form clusters. The center of the graph is occupied by eukaryotic and eukaryotic-like kinases (ePK and ELK). Some *Legionella* kinases (e.g., LegK1–4) are found within and nearby this central cluster. This may indicate a horizontal gene transfer whereby eukaryotic host kinases could have been acquired by the bacteria. In contrast, some *Legionella* kinases are clearly separated from eukaryotic ones in the graph (e.g., Lani_1194, Lcin_0519, Lmor_1975, LepB, AnkK, MavQ, SidJ) which suggests bacterial origin and/or rapid evolution in the pathogen. Finally, others are clustered with atypical host kinases (e.g., ABC1, PI3_PI4_kinase, PIP5K) which may suggest “ancient” kinases present in bacteria and eukaryotes.

Some clusters are eukaryotic- or Metazoa-specific (e.g., Alpha kinases, FAM20), while others appear to be specific to bacteria (e.g., HipA). All the 13 novel families of *Legionella* kinases (see Fig. 5) are at the peripheries of the graph, indicating divergent evolution and arguing against host origin.

From among the 13 novel *Legionella* kinase families, those with most interesting functional implications are discussed in more detail below.

### A kinase that may decorate bacterial secreted factors: Lani_1194

The putative effector kinase Lani_1194 is found in *Legionella anisa* and 15 other *Legionella* species (but not in *L. pneumophila*). *L. anisa* is the second most often isolated *Legionella* species in water samples, following *L. pneumophila*. This species is associated with cases of legionellosis. We can surmise that 6 out of 15*Legionella* species having this protein are human pathogens (*L. parisiensis, bozemanae, jamestowniensis, tucsonensis, jordanis* and *anisa*)^1,48,49^.

**Figure 6:**
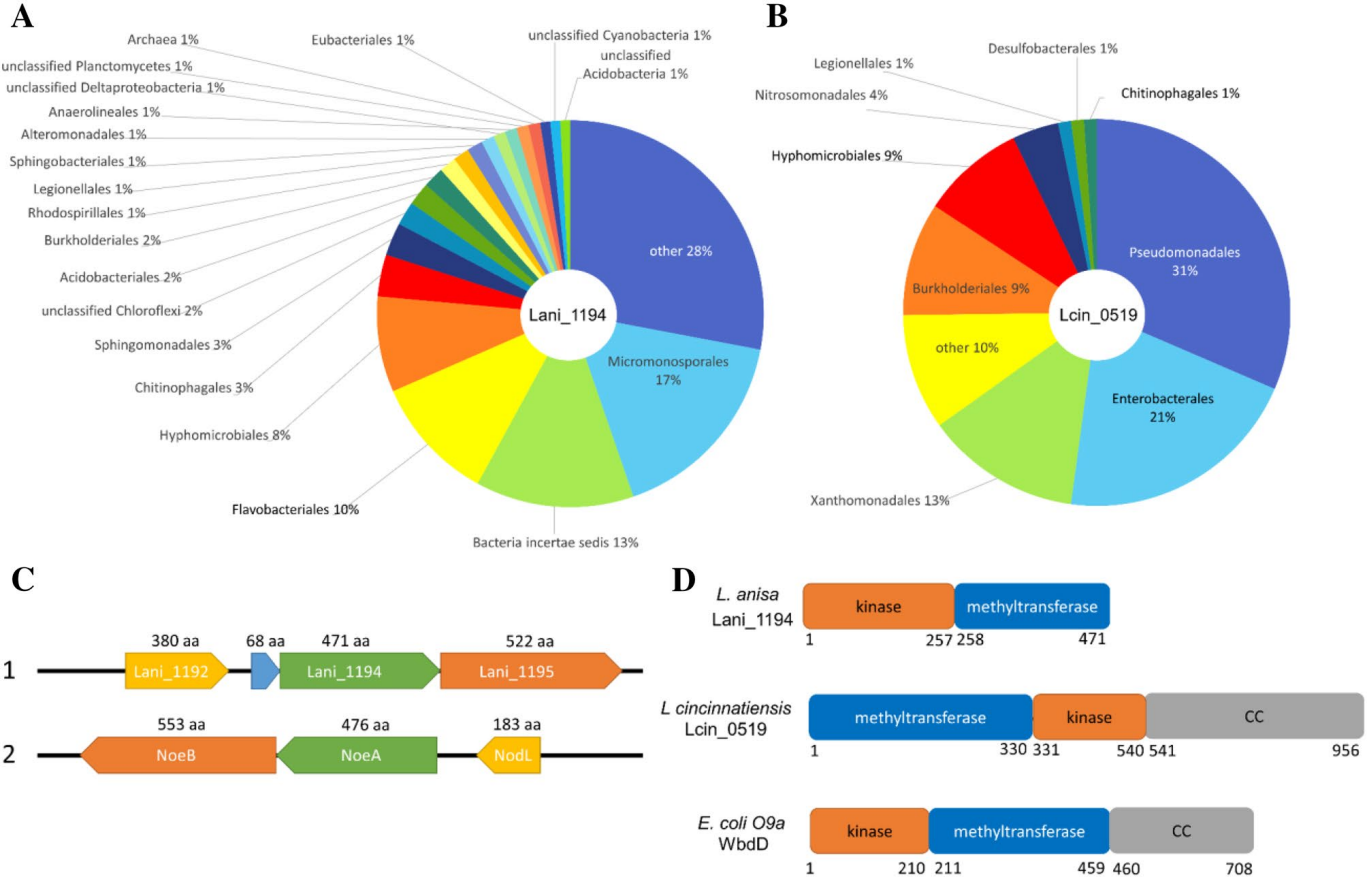
Lani_1194 and Lcin_0519—taxonomic spread, genomic neighborhoods, domain compositions. Organisms with homologs of: (**A**) Lani_1194 and (**B**) Lcin_0519 (found by BLAST search using the kinase domains as queries). Order level shown, or higher if not available. “Others” include taxa containing from 1 to 4 hits (organisms)^28,29^. (**C**) Genomic neighborhood of the protein Lani_1194 (1) and its homolog NoeA (2) in a nodulation-related operon from *Sinorhizobium meliloti*; lengths of encoded proteins shown. Coloring reflects homology. (**D**) Arrangement of structural domains of Lani_1194, Lcin_0519 and WbdD proteins. CC denotes the coiled-coil domain.

In addition to *Legionella*, Lani_1194 homologs are found in 1201 species. The most numerous group here are soil bacteria of the order *Micromonosporales* (*Actinobacteria*), followed by *Flavobacteriales* (*Bacteroidetes*; bacteria of various environments) and soil bacteria, plant root symbionts—*Hyphomicrobiales* order of *Alphaproteobacteria*, e.g., the genera: *Rhizobium*, *Sinorhizobium*, *Mesorhizobium* and *Bradyrhizobium*. Majority of bacteria with Lani_1194 homologs appear to be non-pathogenic, although they are also found in some poorly studied strains of *Escherichia coli*, *Vibrio* and *Clostridium* whose pathogenicity is not yet determined. Also, Lani_1194 homologs are present in 12 species of *Archaea* (Fig. 6A). Among them are species from Gram-negative *Thermoproteota* (thermophilic or hyperthermophilic organisms)^50^ and *Methanoculleus* genus (methanogenic *Archaea*)^51^.

**Figure 7:**
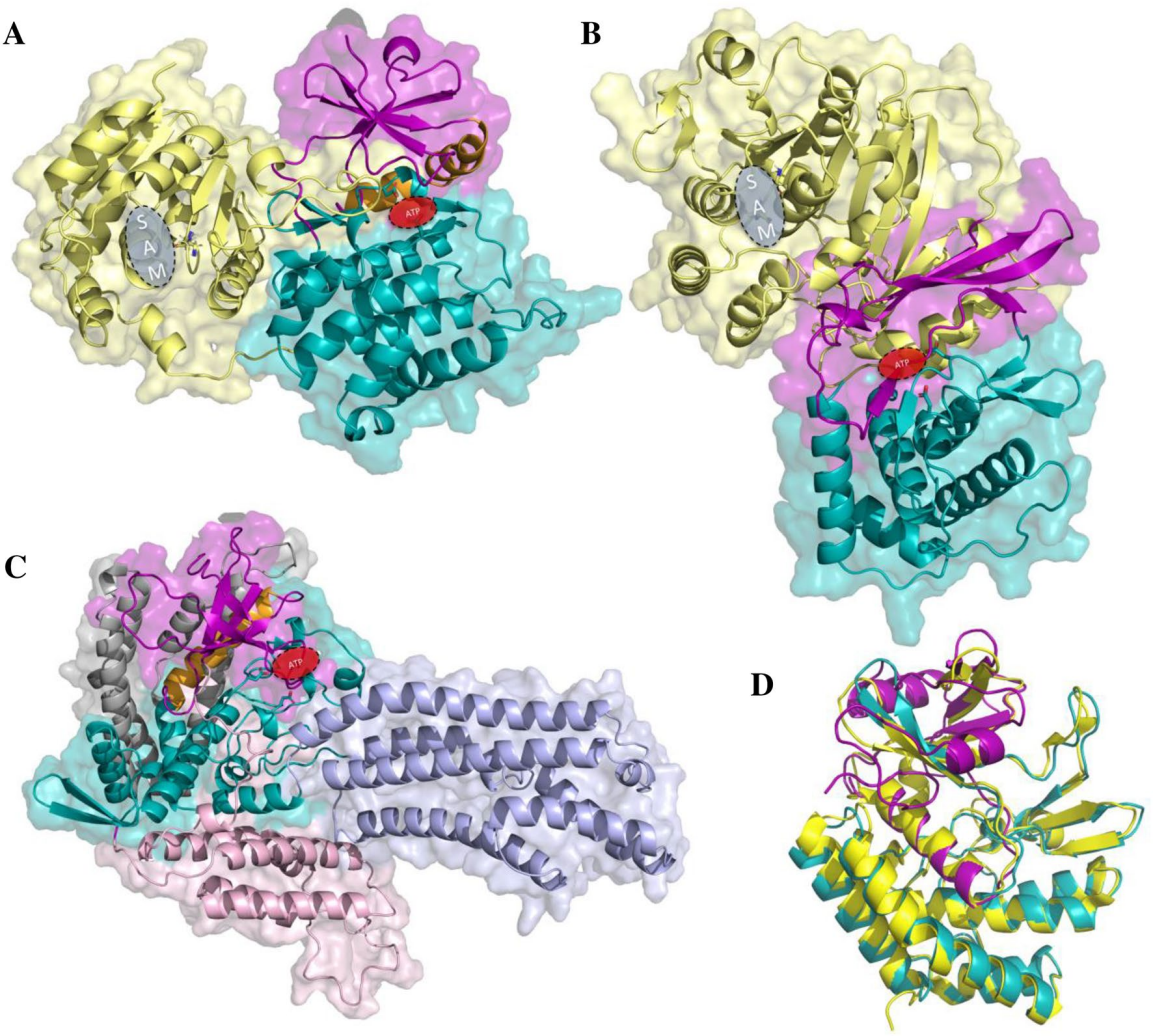
Structures of selected novel kinases. (**A**) Lani_1194, (**B**) Lcin_0519 and (**C**) Lmor_1975 structure models (AlphaFold2). (**D**). Structure comparison of composite HipA models (lpg2379—N-lobe and lpg2380—C-lobe) with lpg2370 structure (PDB:7VKB). Coloring in (**A–C**): Kinase N-lobes: purple, kinase C-lobes: teal, alpha-C helix in the kinase N-lobe: orange, methyltransferase domains: pale yellow. Additional domains in Lmor_1975: helical domain inserted between kinase N- and C-lobes: pink, helical bundle domain; light blue, C-terminal domain: gray. Coloring in (**D**): lpg2370: yellow, lpg2379: magenta, lpg2380: cyan. Residues corresponding to PKA active site D166 and D184 shown in stick representation. A predicted C-terminal coiled-coil region in Lcin_0519, and poorly predicted N-terminal helix in Lani_1194 omitted for clarity. Red ellipses mark the approximate active site region (ATP binding) of the kinase domain. Blue ellipse marks the approximate active site region (S-adenosylmethionine, SAM, binding) of the methyltransferase domain.

Lani_1194 and its homologs have a conserved kinase active site (see Fig. 1) albeit an arginine R38 is most likely the equivalent of the catalytic K72 of PKA. However, neither sequence analysis nor structure model allowed identification of the ion pair glutamate. The aspartate and asparagine residues corresponding to catalytic D166, N171 and D184 of PKA are conserved albeit within atypical sequence motifs (see Fig. 1). Sequence conservation analysis and structure model allow us to delineate the extent of the kinase domain (see Fig. 7A, Suppl. Fig. S8), including a region remotely similar to the ATP-binding glycine-rich-loop.

The Lani_1194 protein also contains a second, easily identifiable domain, the class I S-adenosyl-methionine (SAM) dependent methyltransferase domain (SDM, MTase). Typically, SDMs transfer methyl groups from SAM to a wide range of acceptors, including small metabolites and biological macromolecules, DNA and proteins (e.g., histones) (Suppl. Table S5, Fig. 6D)^52^. According to the AlphaFold structure model, the two enzymatic domains form an extensive interface, with most contacts involving kinase C-lobe (see Fig. 7A).

The kinase—methyltransferase domain architecture is conserved: 94% of approx. two thousand Lani_1194 kinase domain homologs also have the methyltransferase domain.

Analysis of the genomic neighborhoods of Lani_1194 homologs indicated remarkable conservation of immediate genomic neighbors: Lani_1193 and Lani_1195 homologs occur in 65 and 38% of 816 analyzed genomic neighborhoods, respectively. Also, the closest genomic neighbors of Lani_1194 are most often located on the same DNA strand^53^. Such a conserved neighborhood may indicate an evolutionarily conserved functional unit (see Fig. 6C, Suppl. Table S6). Although Lani_1194 is functionally uncharacterized, its homolog and the homolog of its genomic neighbor Lani_1195, NoeA and NoeB (Suppl. Tables S5–S7), belong to an operon of *Sinorhizobium meliloti* which regulates the nodulation of particular *Medicago* plant species by chemically modifying nodulation factors (NFs), signaling molecules secreted by the bacteria to induce host plant to develop symbiosis-allowing root nodules. The biochemical “decoration” of NFs, specific for each bacterial strain and its host plant, occurs in the bacterial cytoplasm before NF secretion and is necessary for recognition of the bacterium as a potential symbiont^54^.

The Lani_1195 and NoeB proteins are predicted to adopt the alkaline phosphatase fold (Suppl. Table S5). The Lani_1192 protein is annotated as O-antigen acetylase, its function is O-acetylation of LPS^55^. A similar function is performed by the NodL protein albeit with a different fold, from the NodL–NoeA–NoeB operon. NodL is also an acetyltransferase responsible for the O-acetylation of sulphated NFs.

NoeA protein, together with NodL and NodB, is possibly involved in the regulation of nodulation through modification of NF signaling molecules in *Rhizobium*^54^. In *Legionella*, the immediate genomic neighbors of the effector Lani_1194 (Lani_1193, Lani_1195) likely encode effector proteins (Suppl. Table S7). The roles of these nodulation gene homologs in *Legionella* infection are not clear but they might be decorating yet unknown signaling molecules secreted by the bacterium into the host cell or may act on the bacterial envelope.

### A kinase that may decorate bacterial outer membrane lipopolysaccharides: Lcin_0519

The novel family of predicted effector kinases Lcin_0519 is found in the human pathogen *L. cincinnatiensis* and five other *Legionella* species^56,57^*.*

Outside the *Legionella* genus, 871 homologs of Lcin_0519 in 333 species were found. The largest groups here are *Pseudomonadales* and *Enterobacteria*. In addition, *Xanthomonadales*, *Burholderiales* (mostly pathogens), *Hyphomicrobiales* (root symbiotic bacteria) and *Nitrosomonadales* (nitrification bacteria) are noticeable. Among the well-known organisms, it is found in some pathogenic human species, such as *Serratia marcescens*, *Klebsiella pneumoniae*, *Vibrio cholerae*, *Burkholderia cenocepacia*, and some known plant pathogens, such as *Pseudomonas syringae* or *Xanthomonas citri* (Fig. 6B).

The Lcin_0519 protein possesses the typical kinase catalytic residues (see Fig. 1, Suppl. Fig. S8), except the ion pair glutamate could not be identified. Indeed, AlphaFold structure model suggests that Lcin_0519 does not have an equivalent of the helix α-C present in most known protein kinases, and the β-sheet of the kinase N-lobe continues into the methyltransferase domain as its central β-sheet (see Fig. 7B).

Similarly to Lani_1194, the Lcin_0519 protein contains a second, easily detected domain, a methyltransferase (see Fig. 7B, Suppl. Table S5). According to the AlphaFold structure model, relative orientation of the two domains is different than in Lani_1194. The inter-domain interface in Lcin_0519 is even more extensive and involves both kinase lobes. This domain architecture is strictly conserved: 96% of proteins with Lcin_0519-like kinase domain also have the MTase domain (Fig. 6D), also common is a coiled-coil domain. Analysis of the co-occurrence of selected genes from close neighborhoods of Lcin_0519 homologs in 493 bacterial genomes (including 8 *Legionella* genomes) shows that 27% of the neighborhoods contain homologs of Lcin_0518 and Lcin_0520 while in 17% of neighborhoods there are also homologs of Lcin_0517^53^ (Suppl. Table S6).

The Lcin_0518 protein is annotated as an ABC transporter of LPS O-antigen (Wzt), Lcin_0517—as an LPS transport system permease (Wzm) and Lcin_0520—as a glycosyltransferase (GTase). Together, these proteins in *Aquifex aeolicus* (Wzt, Wzm and GTase) secrete the complete O-antigen across the inner membrane for ligation to the LPS core^58^.

Thus, also in *Legionella,* the Lcin_0519 kinase-MTase and its genomic neighbors can be predicted to be related to the modification of the bacterial outer membrane, e.g., Lcin_0519 might modify LPS through phosphorylation and methylation.

The protein domain composition of Lcin_0519 is reminiscent of a known enzyme, WbdD protein from *E. coli O9a*. WbdD has kinase, methyltransferase and CC domains (Fig. 6D). WbdD proteins are strain specific and regulate chain termination and length modifications of O-antigen^59^. However, WdbD and Lcin_0519 are clearly different, remotely related, kinase-MTase families. Although Lcin_0519 is annotated bioinformatically as an effector, it has not to our knowledge been studied experimentally. Thus, it can be speculated that Lcin_0519 may be not an effector, but indeed a “household” enzyme involved in the synthesis of LPS. In *Legionellas* it is known to be unique in comparison to most Gram-negative bacteria, highly variable between strains and species, and essential for infectivity^60,61^. Lcin_0519 may be therefore acting on the *Legionella* envelope and contributing to pathogenicity by adjusting envelope-host cell interactions to the requirements of the infection stage.

### A kinase with a large internal insertion: Lmor_1975

Another unusual, predicted effector kinase was found in *L. moravica.* This protein, Lmor_1975, has homologs in only a few other closely related species (mainly *Legionella*, some potentially pathogenic to humans)^56^.

Although sequence analysis (HHpred) detected Lmor_1975 similarity only to the C-lobe of LepB kinase, using sequence conservation and structure prediction we have identified equivalents of ion pair Lys and Glu in the N-lobe. Sequence analysis suggested, and AlphaFold structure model showed that Lmor_1975 has a large alpha-helical insertion between N-lobe and C-lobe, consisting of approx. 150 residues (see Fig. 7C, Suppl. Fig. S8). This is reminiscent of an insertion of approx. 80 amino acids found in atypical FAM69/DIPK kinases from Metazoans. The insertion in FAM69 contains an EF-hand calcium ion binding motif, located close to the ATP pocket between the N-lobe and the C-lobe and predicted to modulate kinase activity^62^. In Lmor_1975, the large insert and additional helical domains in the C-terminal region of the protein (see Fig. 7C) suggest a layer of regulation of kinase activity, possibly by interaction with intracellular structures or molecules.

The sequence similarity graph analysis located Lmor_1975 close to PI3K families: OpiA/AnkK^18^ and MavQ^19^, which suggests it may be a PI kinase (see Fig. 4).

The large helical insertion between N-lobe and C-lobe clearly obscures structural similarity to the PKL fold. The very weak similarity of Lmor_1975 to known kinases observed both in sequence and structure searches underscores the difficulty of recognizing distant homology in cases of large inserts within structural domains.

### A likely “composite” HipA protein kinase formed from the products of lpg2378, lpg2379 and lpg2380 genes

Analyzing the “known” kinase effectors, we noticed a peculiar “composite” HipA kinase in *L. pneumophila.* HipA kinases play a very important role in stress response mechanisms of *E. coli* and many other Gram-negative bacteria by inducing a dormant state termed persistence. In *E. coli*, HipA is part of a toxin-antitoxin type system also including its genomic neighbor, the HipB antitoxin^63,64^. HipA phosphorylates glutamyl-tRNA synthetase, which results in inhibition of protein synthesis and growth arrest^64,65^. The activity of HipA is inhibited by binding to HipB and by HipB acting as a transcriptional autosuppressor of the hipBA operon^64^.

**Figure 8:**
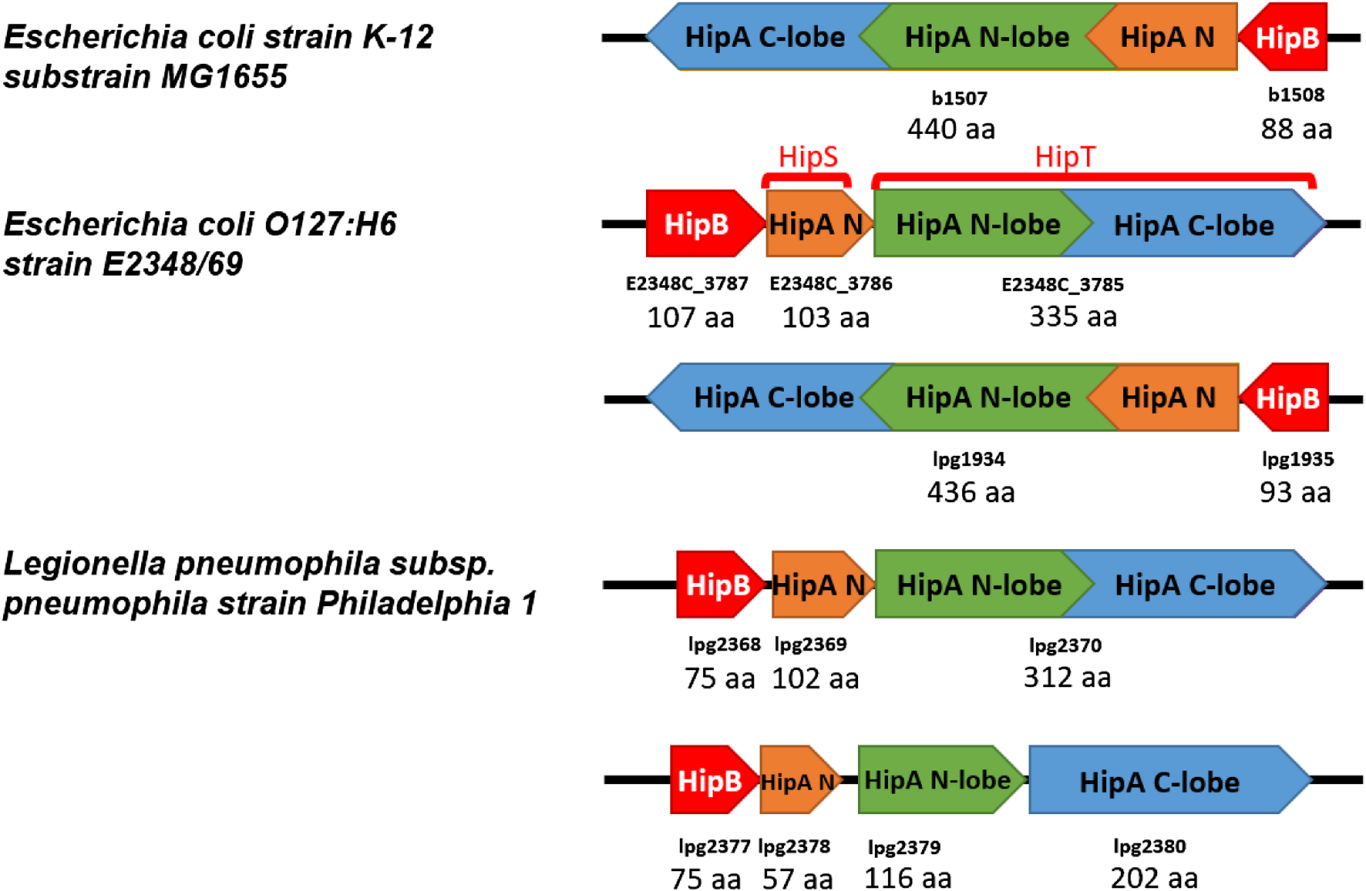
HipA-like modules in different bacteria. Gene loci names and protein lengths are shown below the gene diagrams. The colors represent HipB (red) and the subdomains of HipA (HipA-coupled N-terminal domain—orange, kinase N-lobe—green, kinase C-lobe—blue). The *L. pneumophila* protein lpg1934 (not shown) has the same gene/domain arrangement as *E. coli* K12 HipA.

In *L. pneumophila*, the putative “composite” kinase is encoded by two adjacent genes whose protein products together may form the complete HipA-type kinase domain (Fig. 8). Thus, lpg2379 encodes the kinase N-lobe and lpg2380 — the C-lobe. This likely indicates a gene fission phenomenon^66^.

In the *L. pneumophila* genome near the lpg2379 and lpg2380 genes (8 kbp away) lies the lpg2370 gene which encodes a complete kinase domain of HipA type. The lpg2370 protein shares approx. 90% sequence identity with the lpg2379-lpg2380 pair, and consequently very high structural similarity (see Fig. 7D), which may indicate a recent duplication of an ancestral HipA-like gene and splitting of one the copies. The duplicated arrangement involving homologs of lpg2379-lpg2380 and lpg2370 genes is found only in 35 strains of *L. pneumophila* (e.g., Philadelphia-1, Burlington 1 (D-7841)).

Both lpg2370 and lpg2380 are predicted to be T4SS effectors (Suppl. Table S4).

Next to lpg2379-lpg2380 and to lpg2370 there are also genes encoding another element of the “classic” HipA protein, the N-terminal “HipA-coupled” domain (lpg2369 and lpg2378, respectively), responsible for dimerization during DNA binding^63^ (see Fig. 8). Further, both HipA-like proteins are accompanied in the genome by homologs of the HipB antitoxin, lpg2368 and lpg2377, respectively. Interestingly, both HipB-like proteins are weakly predicted to be effectors. It remains to be tested if indeed *Legionella* delivers HipA-like kinase(s) to the host cell cytoplasm, and whether these effectors are accompanied by N-terminal subdomains and HipB suppressors.

Yet another *L. pneumophila* protein, lpg1934, appears to be a typical HipA (28% identity to *E. coli* HipA). It has a full kinase domain, an N-terminal “HipA-coupled” domain in one protein, and it’s not recognized as an effector (Suppl. Table S4).

The lpg2379 and lpg2380 genes overlap by 40 nucleotides, a kind of overlap observed often in prokaryotic genomes. Also, lpg2378 and lpg2379 genes lie in the + 1 reading frame while lpg2380 lies in the + 2 reading frame. The fact that lpg2379 and lpg2380 genes lie in two different reading frames argues against separation of these genes being the result of gene misprediction or sequencing error^67^.

In an analogy to our observation, a protein from the HipA family was recently discovered in *E. coli O127*, split into two proteins encoded by distinct genes: a kinase domain (HipT gene) and an N-terminal HipA-coupled domain (HipS gene). Recently, it has been shown that lpg2368–lpg2369–lpg2370 act as a HipBST toxin-antitoxin system similar to that in *E. coli*^68^. The lpg2379-lpg2380 pair is another case of an elaborate HipA module and an example of how gene fusion, fission and duplication shape and create new cellular signals^69,70^. The possibility that *Legionella* employs the purely bacterial HipA family to manipulate eukaryotic signaling is particularly interesting, given HipA have evolved in the context of bacterial intracellular signaling.

Further, the composite kinase may offer a yet unknown layer of kinase regulation by assembly of a functional enzyme from subunits from separate polypeptide chains.

## Conclusions

In this bioinformatic analysis of 41 *Legionella* species, we cataloged 112 protein kinase-like Legionella Orthologous Groups (LOGs) within 29 families, of which 13 families are novel. We have discussed in detail sequence/structure features and proposed functional predictions for three novel families and a putative new composite HipA kinase. The novel PKL families identified by sequence searches were confirmed by artificial intelligence-based structure predictions.

Two novel families, Lani_1194 and Lcin_0519, were found to occur far beyond *Legionellas*. This introduces an intriguing prospect of related enzymatic machinery being used for different purposes in different biological scenarios, i.e., for nodulation-related signaling between rhizobial bacteria and plant hosts, and for rewiring intracellular signaling in amoebas and animals infected by *Legionellas.* Although literature evidence suggests most *Legionella* effectors act on host cell molecules or on each other, acting on bacterial own cell envelope can also be relevant for infection^60,61^.

An inherent limitation of the present study is the fact that these functional predictions rely on literature data available for homologs. Nevertheless, this makes the novel kinase-like families even more attractive subjects for experimental studies. In a rather unlikely case the effector predictions for the novel families are wrong, these families still may be attractive as targets of a therapeutic intervention, because even if not delivered to the host cell they are likely to perform roles important for the pathogen’s survival.

*Legionella* kinomes are rich in effector kinases in addition to their sets of “household” kinases. This indicates their adaptation to different hosts—mostly *Protozoa*, but also higher eukaryotes. The most studied *Legionella* species — *L. pneumophila* has a set of genes for both infecting various *Amoebae* and macrophages in the human lung. Some of these kinases, such as LegK1-4, structurally and sequentially closely resemble eukaryotic kinases, perhaps having been “hijacked” by the way of gene transfer from eukaryotes and evolutionarily adapted. Others, while retaining the PKL fold, appear to be very distantly related to known kinases, which obscures their evolutionary origin, likely due to high evolutionary pressure.

Thus, we have created a catalog of *Legionella* (pseudo)kinases, available at http://bioinfo.sggw.edu.pl/kintaro/ thanks to the comprehensive analysis of the pan-proteome of 41 species of this genus. The discovery of these kinases may aid in developing new approaches to fight these pathogens. Moreover, these novel families are often found in other pathogens of animals and plants. Thus, the survey of *Legionella* pan-kinome presented herein offers starting points into studies of this pathogen’s infection toolbox, but also a broader perspective on the ingeniousness of nature in diversifying, developing and repurposing the successful kinase-like superfamily.

## Materials and methods

### Search strategy

The general approach used in this work to search for novel kinase-like families was described recently^71^. Briefly, the screen for novel kinase-like proteins starts with a set of protein sequences (here *Legionella* proteins set) where redundancy is reduced and representative sequences are split into fragments. In the next steps algorithms for remote homology detection are used (FFAS, HHpred) for searching and validating similarity to kinases. Additionally, for candidate kinase-like proteins, three-dimensional structure models are built and compared with known kinase structures.

### Sequence data

Protein sequence data for *Legionella* effectors was provided by the article by Burstein et al.^25^.

### Clustering

Due to the large size of the sequence data, the sequences were clustered by sequence identity using the CD-HIT algorithm^31^. Two clustering thresholds were used: 90% and 50% sequence identity. This reduces the load on the processor.

### Splitting sequences

Sequences were split^32^ into 300 aa length with overlap of 100 aa.

### Remote homology detection

For distant similarity prediction to PKL families three methods were used, the profile-profile alignment and fold recognition algorithm — FFAS^33^ (COG^72^, Hsapiens^73^, PDB^74^, SCOP^75^, Pfam^76^ databases); homology detection and structure prediction—HHpred and similar HHsearch pipeline that uses hidden Markov model HMM-to-HMM comparison^36^ (PDB^74^, SCOP^75^, Pfam^76^ databases); and a similar method Phyre2, which additionally models 3D structure of query and compares it with 3D models library^37^. Standard parameters were used, however both significant hits and those not formally significant were taken into account.

### Multiple sequence alignments and sequence logos

Novel families were collected using BLAST (NR, E-value = 1e−4)^28–30^ and aligned using the MAFFT^77^ algorithm with default settings. Next, the sequence logos were prepared using the WebLogo algorithm^27^. The WebLogo program generates the sequence logos based on the multiple sequence alignments. Here, the alignments are processed with an in-house script that removes the columns containing gaps in the “master” sequence.

Secondary structure prediction was performed by Jpred4^38^.

### Structure modeling and comparison

Novel PKL-like structures were modeled with use of RoseTTAFold^39^ and AlphaFold2^40^ (the best models have been selected). Comparisons of structures were performed using FATCAT^41^ and Dali^42^ servers.

### Visual clustering of families (analysis of sequence similarity relations between families)

To visualize clusters of protein kinase families, the cluster of sequences (CLANS) algorithm^47^ was used with the BLOSUM62 scoring matrix and extraction of BLAST hits up to E-value of 1. The set of sequences was collected as follows:

- Newly predicted protein kinase families collected by BLAST (NR database, E-value = 1e−4)^28–30^ and clustered by CD-HIT at 50% sequence identity—Lani_1194. Lcin_0519, or at 99% sequence identity—Lani_2844, Lfee_0407, LLO_1015, LLO_2159, Lmor_1975, lpg1316, lpg1925, Lsai_0337, Lqui_0983, Llan_0165, Lspi_2187)^30^.
- Families of PKinase clan from the Pfam database: APH_6_hur (rp15 sequence set), APH (seed sequence set), Choline_kinase (seed), CotH (seed), DUF1679 (seed), DUF2252 (rp15; flipped N-lobe and C-lobe), DUF4135 (rp15), DUF5898 (rp15), EcKL (seed), Fam20C (seed), Frukosamin_kin (seed), FTA2 (rp35 sequence set), Haspin_kinase (seed), HipA_C (seed), Ins_P5_2-kin (seed), IPK (seed), IucA_IucC (seed), Kdo (seed), Kinase-PolyVal (rp35), Pan3_PK (seed), PI3_PI4_kinase (rp15, clustered at 40% sequence identity), PIP49_C (seed), PIP5K (seed), Pkinase_fungal (seed), Pkinase (rp15, because the rp15 set is very large, it was clustered at 25% identity level, and sequences longer than 300 residues were selected), PK_Tyr_Ser_Thr (seed), Pox_ser-thr_kin (rp15), RIO1 (seed), Seadorna_VP7 (rp75), UL97 (rp15), WaaY (rp55), YrbL-PhoP_reg (rp35), YukC (rp35), families not yet included in PKinase clan, but having PKL fold (LepB_N (rp55 sequence set), FAM198 (rp15), SelO (seed))

Other proteins with predicted or known fold similar to PKL not included yet in Pfam database (collected by BLAST, NR, E-value = 1e−4) — OpiA/AnkK/LegA5, HopBF1, lpg1924/LegK7, MavQ, NleH–OspG, SidJ, Lsha_0263 (XopC2)^30^. All the families were manually curated and corrected (domains were extended when they appeared not to include full kinase-like structural domains). From Lmor_1975 and Lsha_0263, helical inserts were removed.

### Substrates of secretion systems

Substrates of secretion systems were predicted with use of SignalP6.0^44^, EffectiveDB (EffectiveT3, T4SEpre, EffectiveCCBD, EffectiveELD)^45^ and BastionX^46^. All programs were used with default settings.

### Coiled-coil domains

Coiled-coil domain was predicted by DeepCoil^78^.

### Transmembrane helices

Transmembrane helices were predicted with use of TMHMM^79^.

### Phylogenetic trees and species heatmap

Phylogenetic tree of *Legionella* strains was adapted from the article by Burstein et al.^25^. Heatmap of the number of PKL genes was clustered by hierarchical clustering (Manhattan method; single linkage) to see similar arrangements of genes in *Legionella* species.

For kinase-like family phylogenetic trees, multiple sequence alignments were done using the structure alignment program mTM-align^80^. Where no experimental structures were available (e.g., for the novel kinase families), structure models were built using AlphaFold. Alignment trimming was performed using ClipKit^81^ and manually corrected. The phylogenetic tree was built using the MEGA program (default settings) using ML method and bootstrapping = 500^82^. For the PI3-PI4 kinase-like tree, all human representatives were used while amoeba sequences were clustered at 30% sequence identity threshold (cdhit). For the eukaryotic-like kinase tree, human sequences were clustered at 30%.Phylogenetic trees visualization was done in ITOL^43^.

### Potential horizontal gene transfer analysis

For this purpose, we use CLANS^47^ analysis (parameters: BLOSUM62 scoring matrix; extraction BLAST HSP’s up to E-values of 1) with kinomes of *Legionella* and its hosts (*Homo sapiens*, *Dictyostelium discoideum* and *Acanthamoeba castellanii*. CLANS analysis clusters similar sequences into groups.

Taxonomic distribution analysis of homologs was done using BLAST^28–30^. The numbers of bacterial and eukaryotic homologs of *Legionella* eukaryotic-like kinases were compared. In cases where the number of eukaryotic homologs of a *Legionella* ELK is significantly larger than the number of bacterial homologs, an eukaryote-to-bacteria horizontal gene transfer can be hypothesized.

## Supplementary Information

Supplementary Information 1.

Supplementary Information 2.

Supplementary Information 3.

Supplementary Information 4.

Supplementary Information 5.

Supplementary Information 6.

Supplementary Information 7.

Supplementary Information 8.

Supplementary Information 9.

Supplementary Information 10.

Supplementary Information 11.

Supplementary Information 12.

Supplementary Information 13.

Supplementary Information 14.

Supplementary Information 15.

Supplementary Legends.

## Data Availability

The following information was supplied regarding data availability: Raw data (including PDB files for protein structure models) are available in the Supplemental Files. Sets of aligned representative sequences of *Legionella* (pseudo)kinase families are available from the online database at http://bioinfo.sggw.edu.pl/kintaro/.
