# Supplementary figures and images for: Pan-kinome of Legionella expanded by a bioinformatics survey
Source: Sci Rep. 2022 Dec 16;12:21782. doi: 10.1038/s41598-022-26109-x (PMC9758233; doi:10.1038/s41598-022-26109-x)

Lani\_1194 15-192

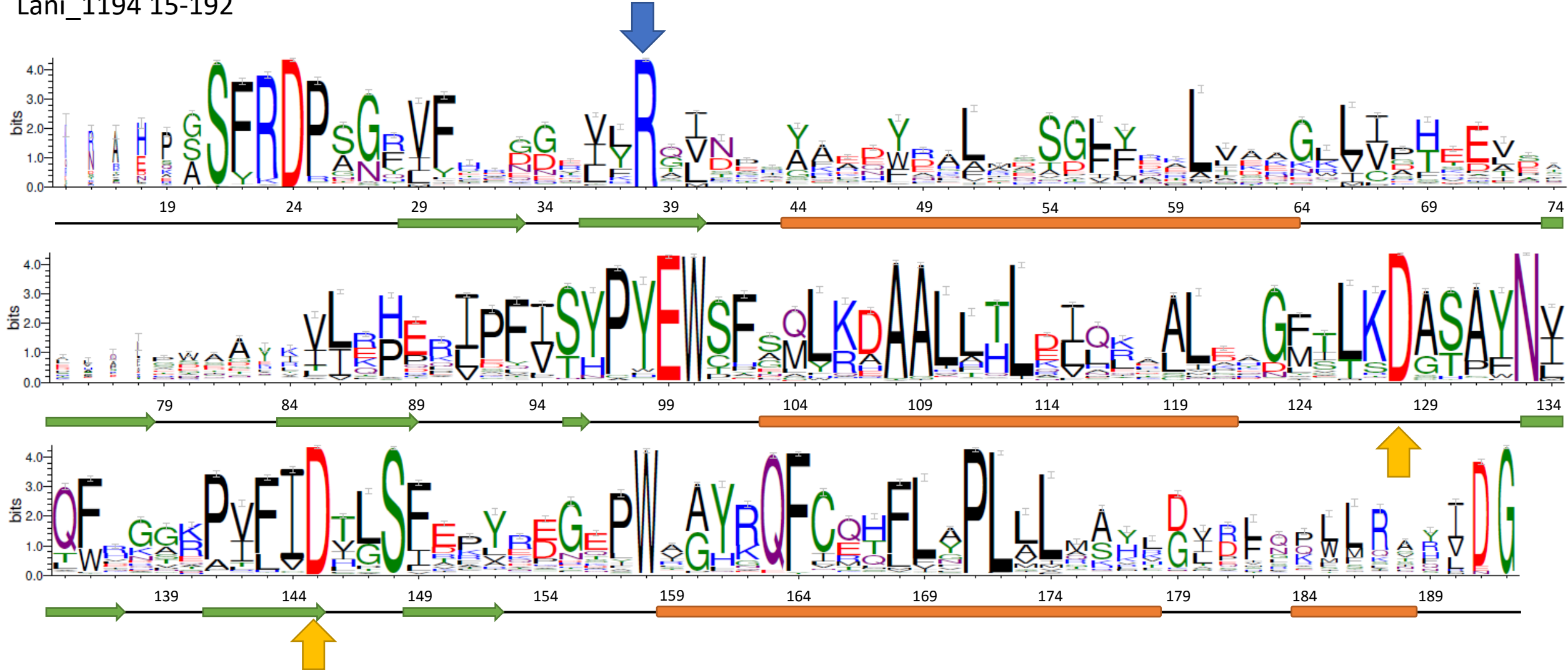

Lani\_2844 20-248

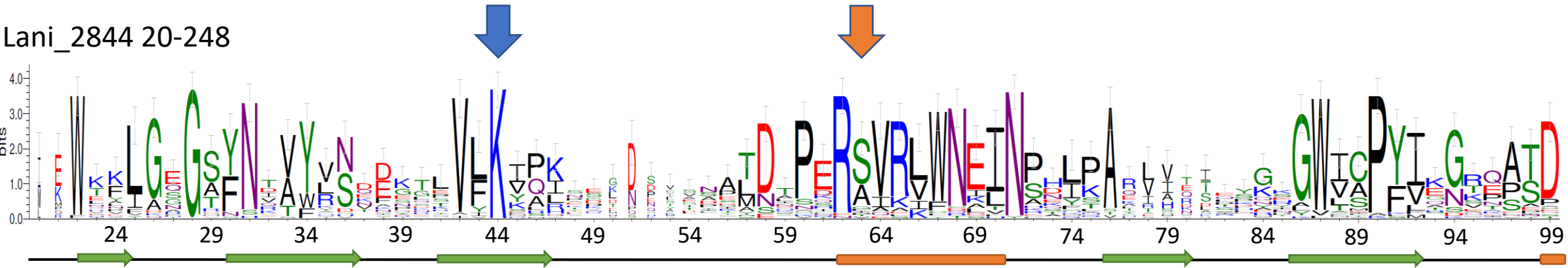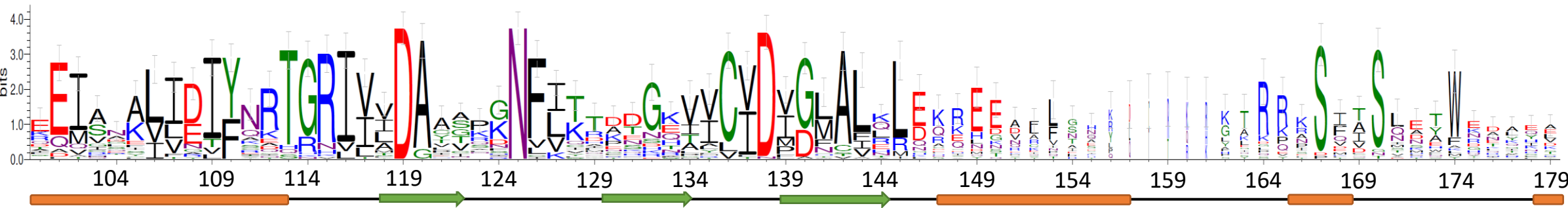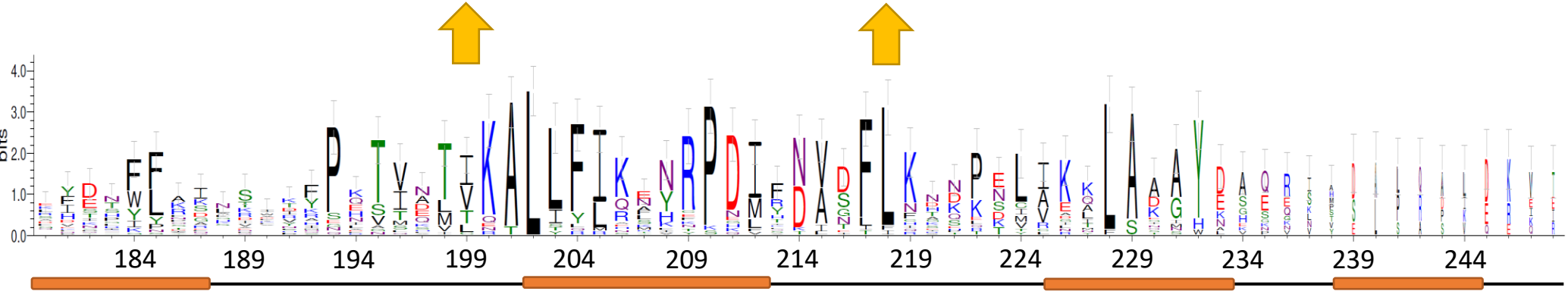

Lcin\_0519 304-543

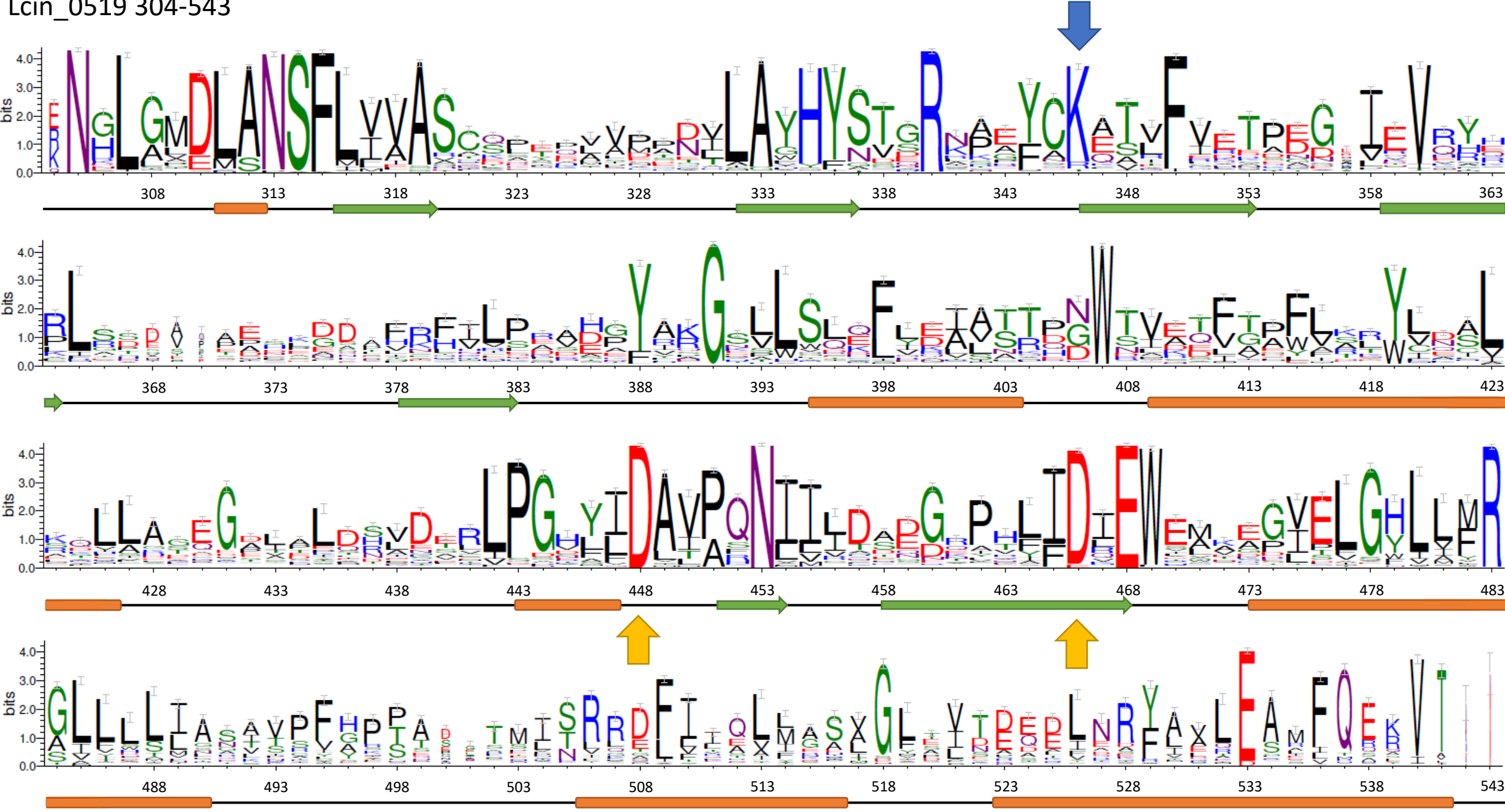

Lfee\_0407 4-205

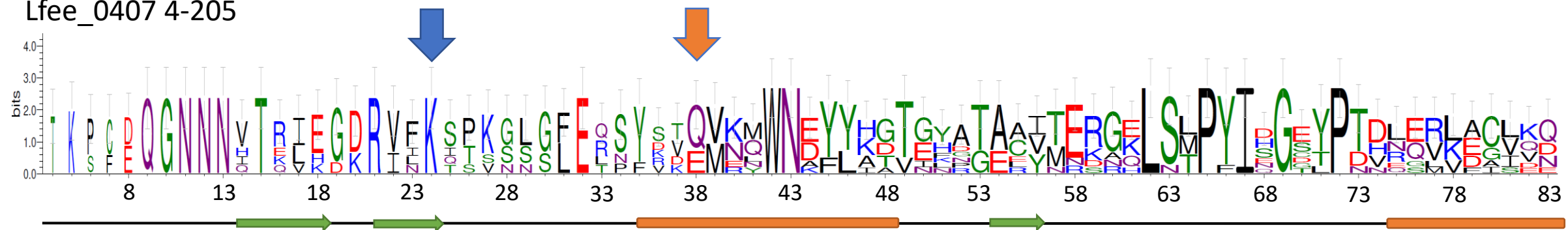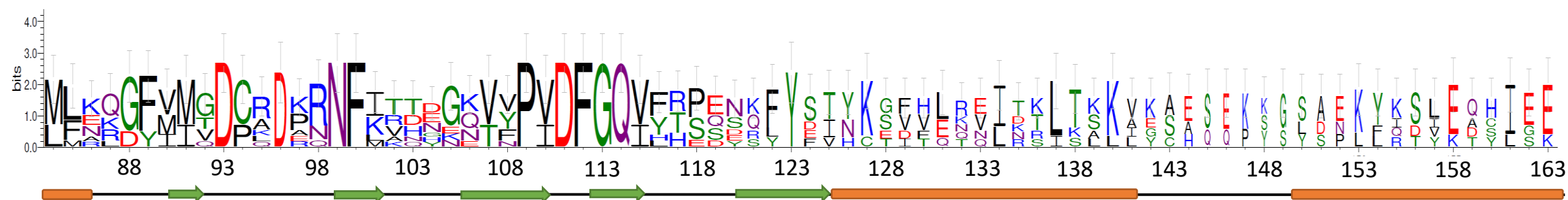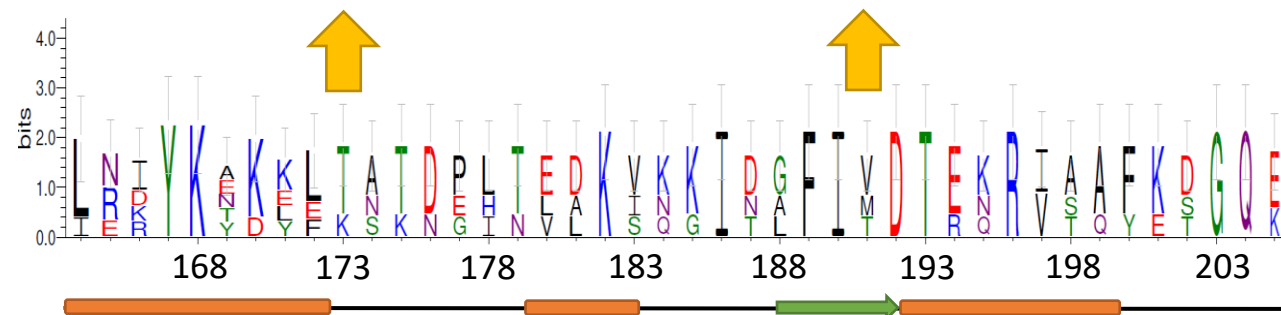

Llan\_0165 13-254

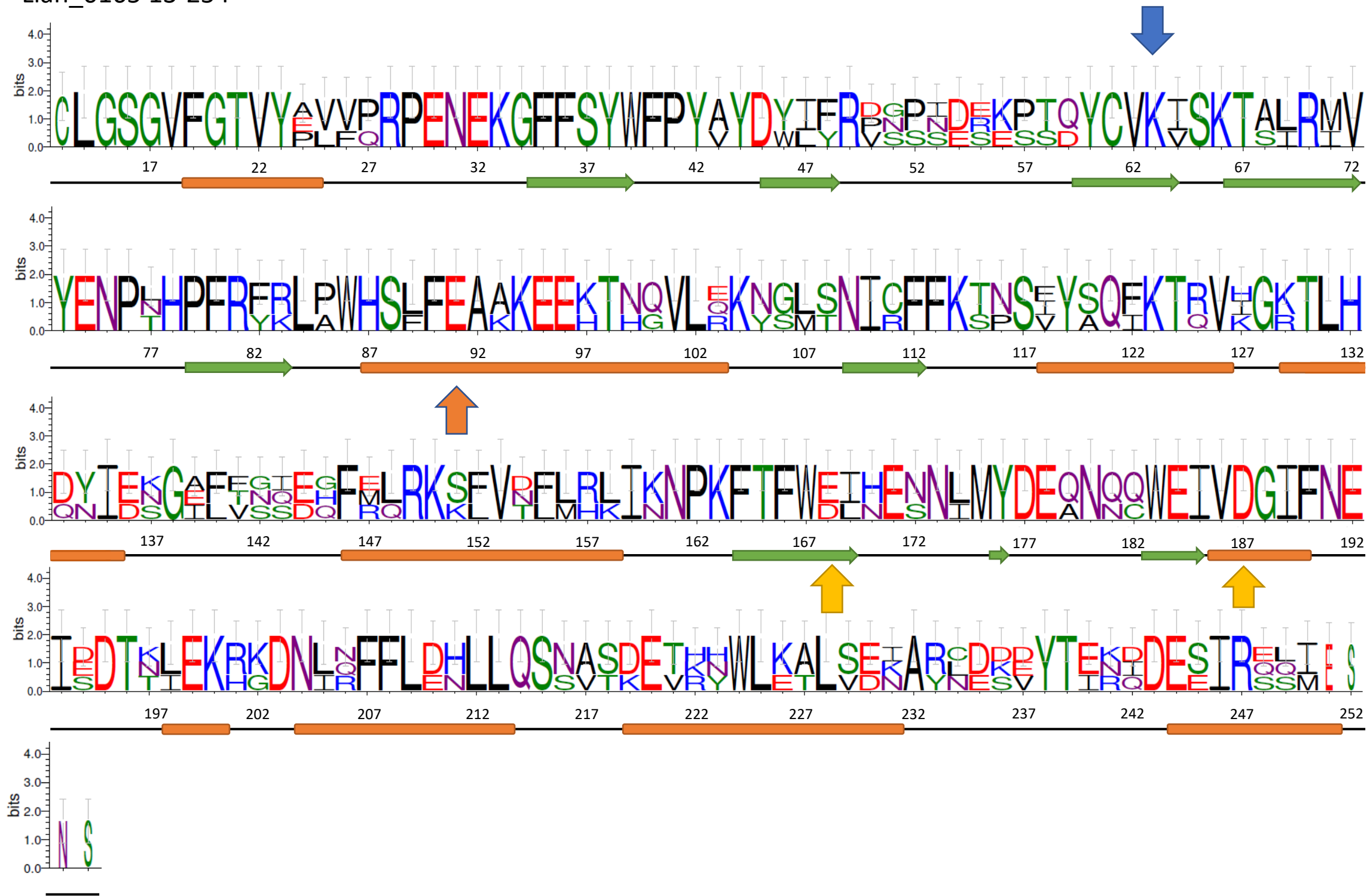

LLO\_1015 1-303

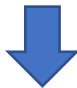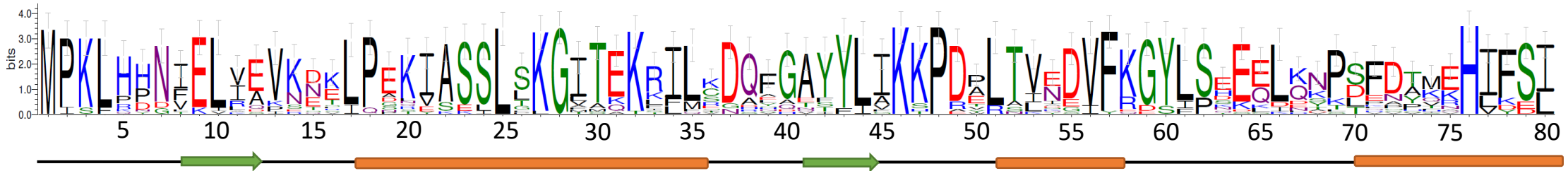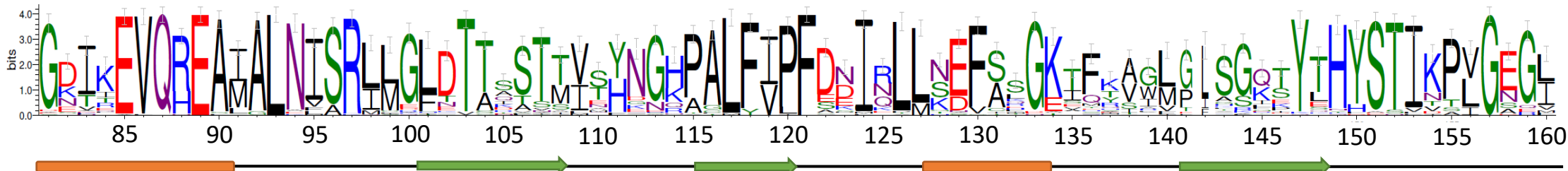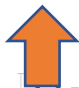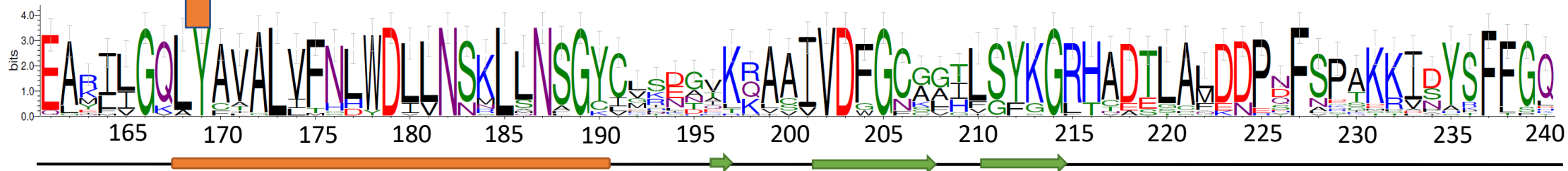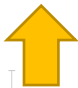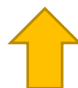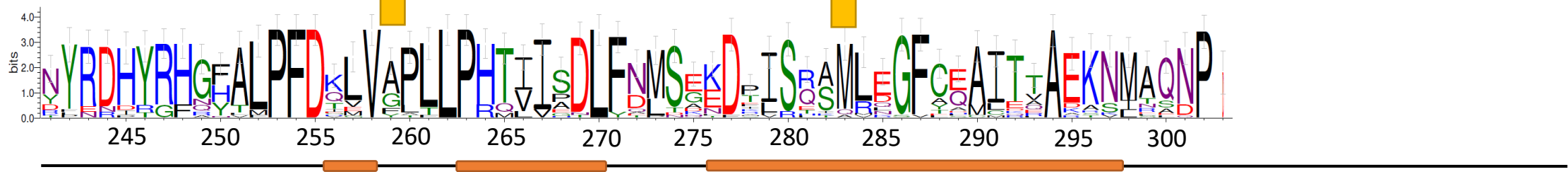

LLO\_2159 194-434

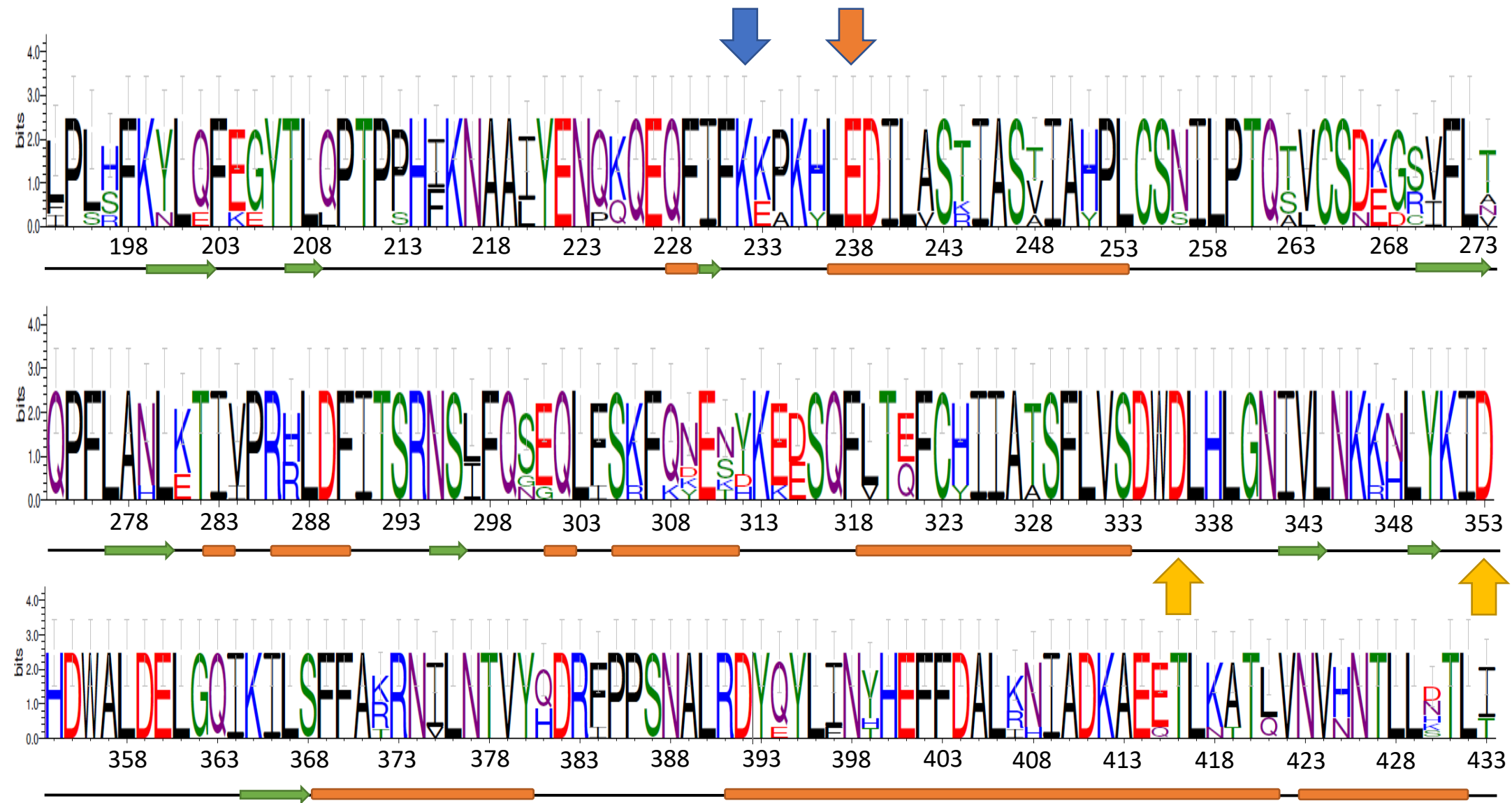

Lmor\_1975 1-420

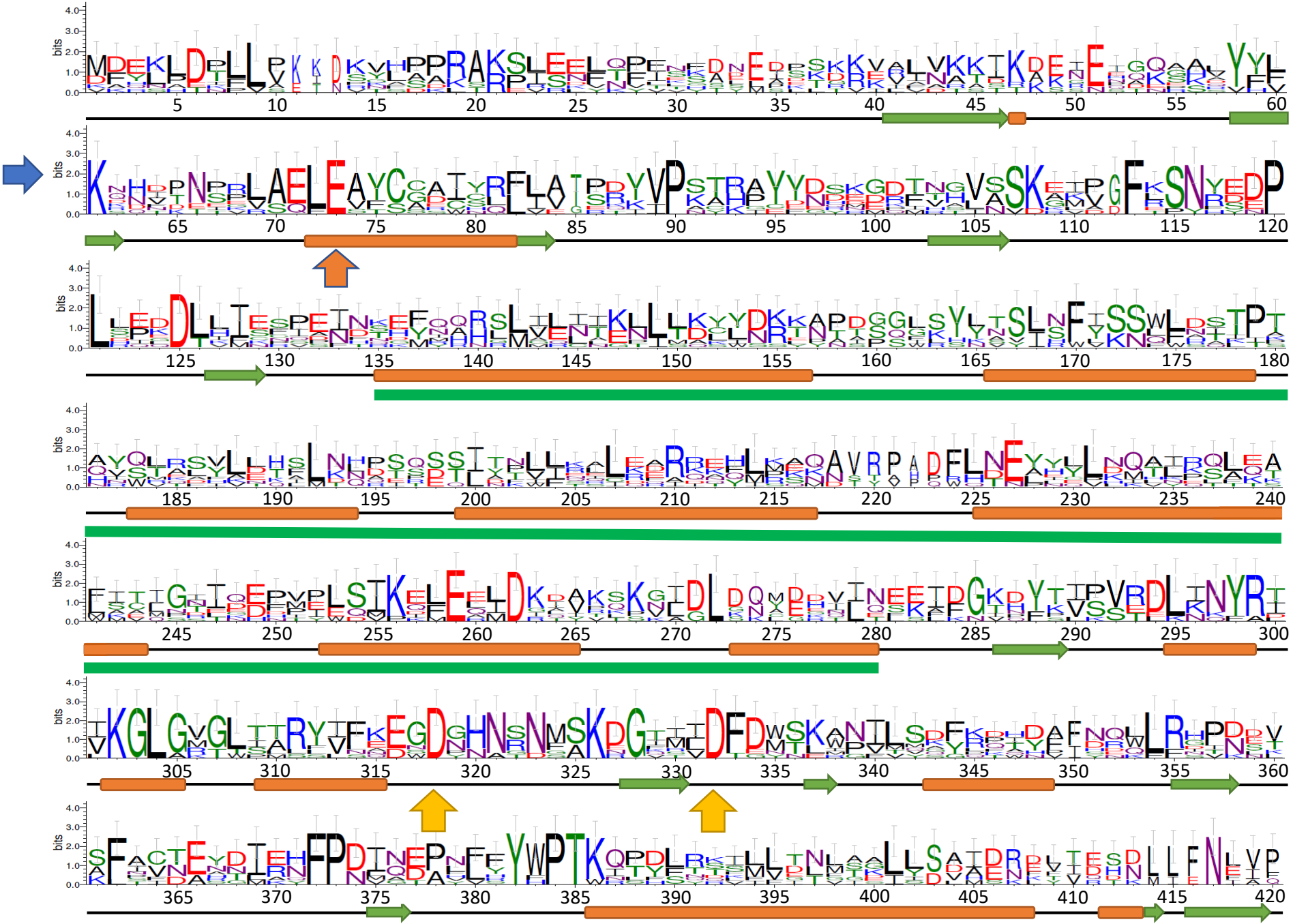

lpg1316 69-311

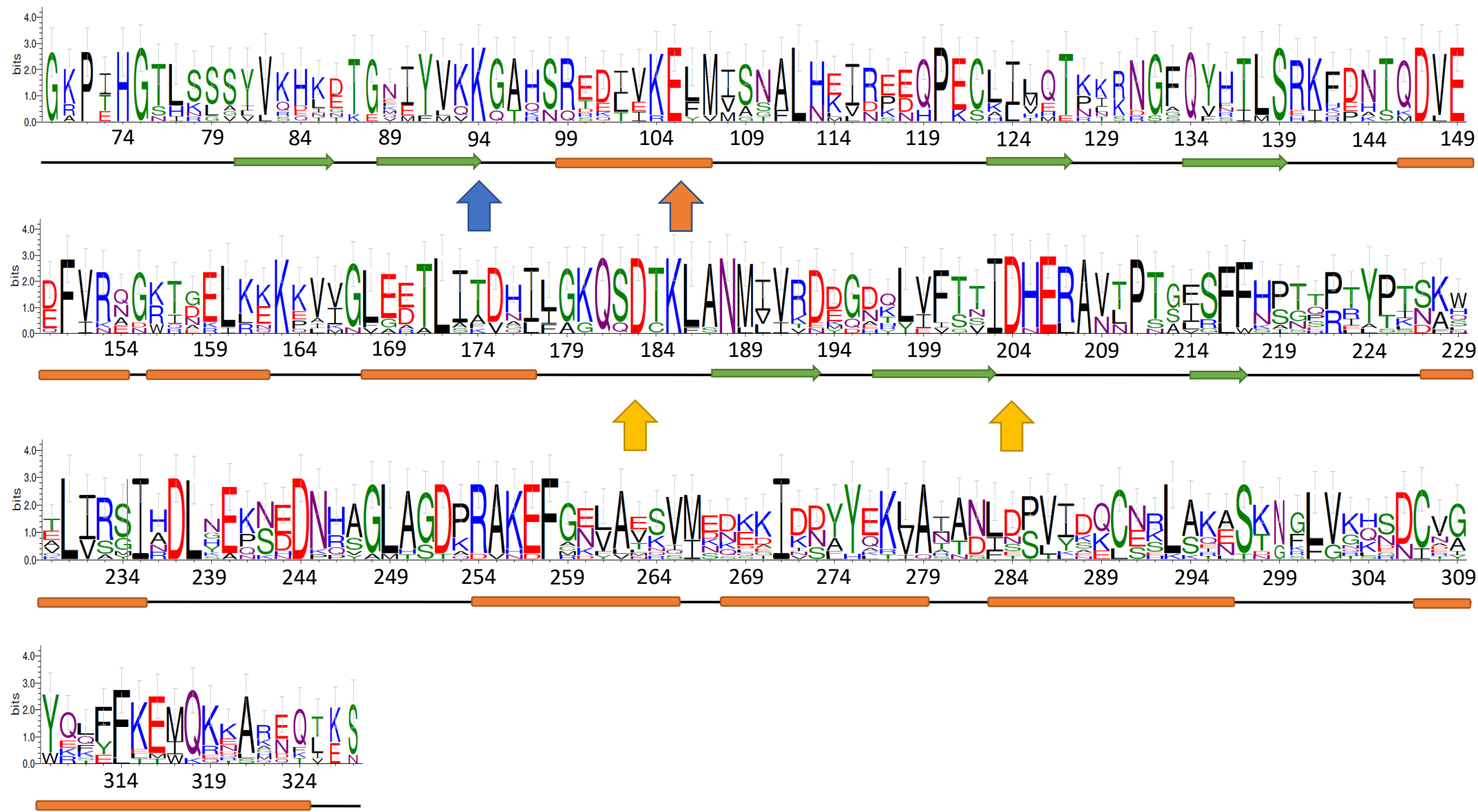

lpg1925 198-525

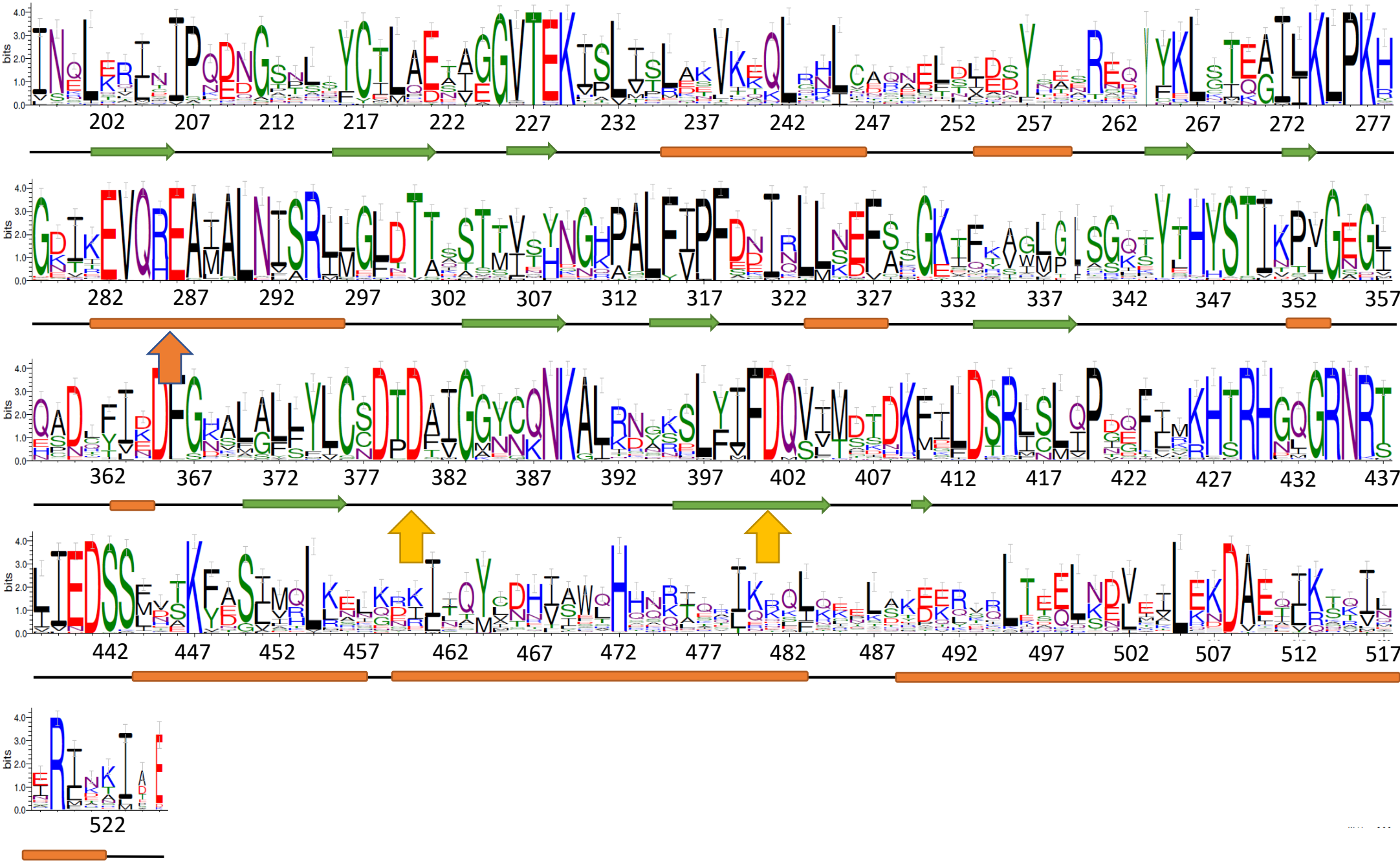

Lqui\_0983 1-248

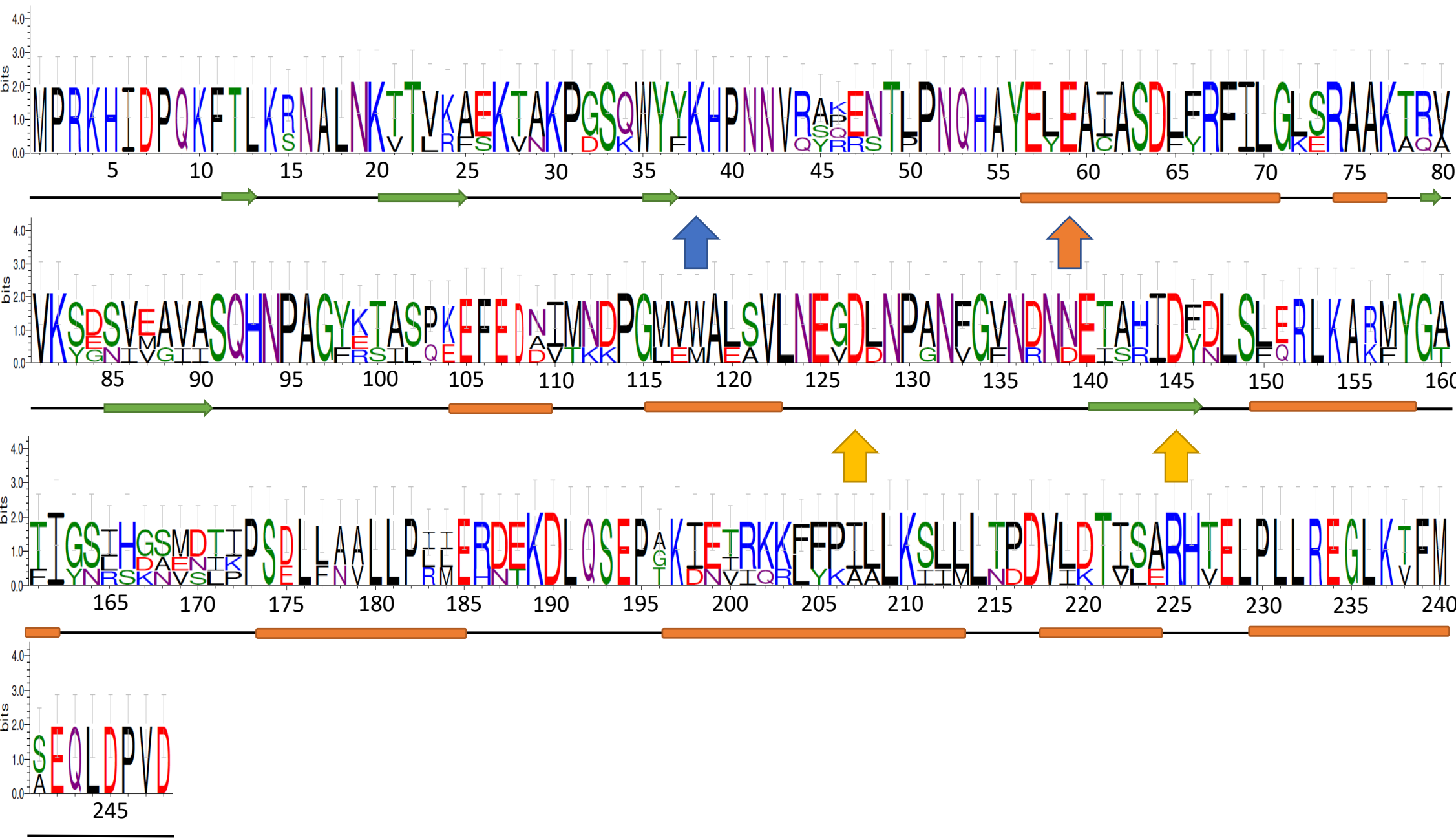

Lsai\_0337 1-231

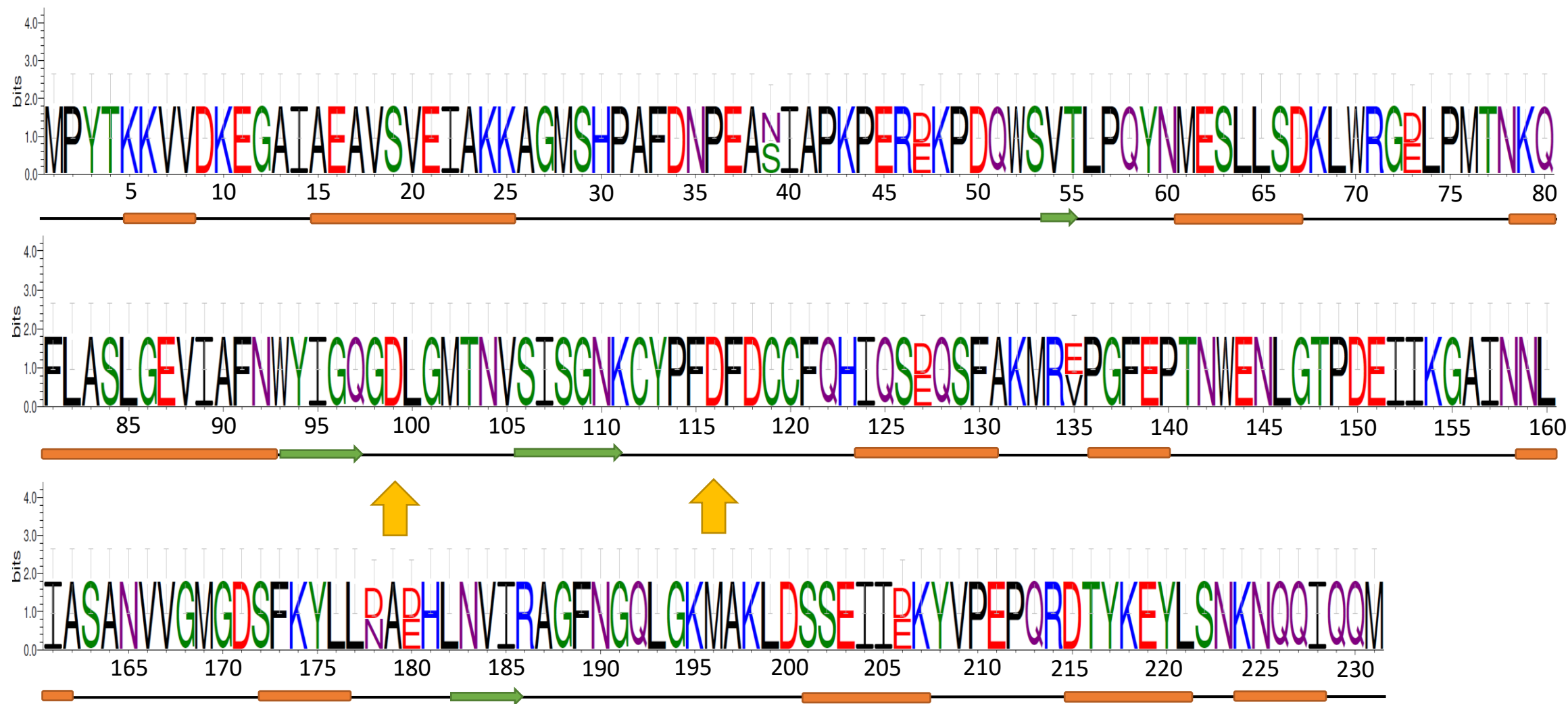

Lspi\_2187 115-352

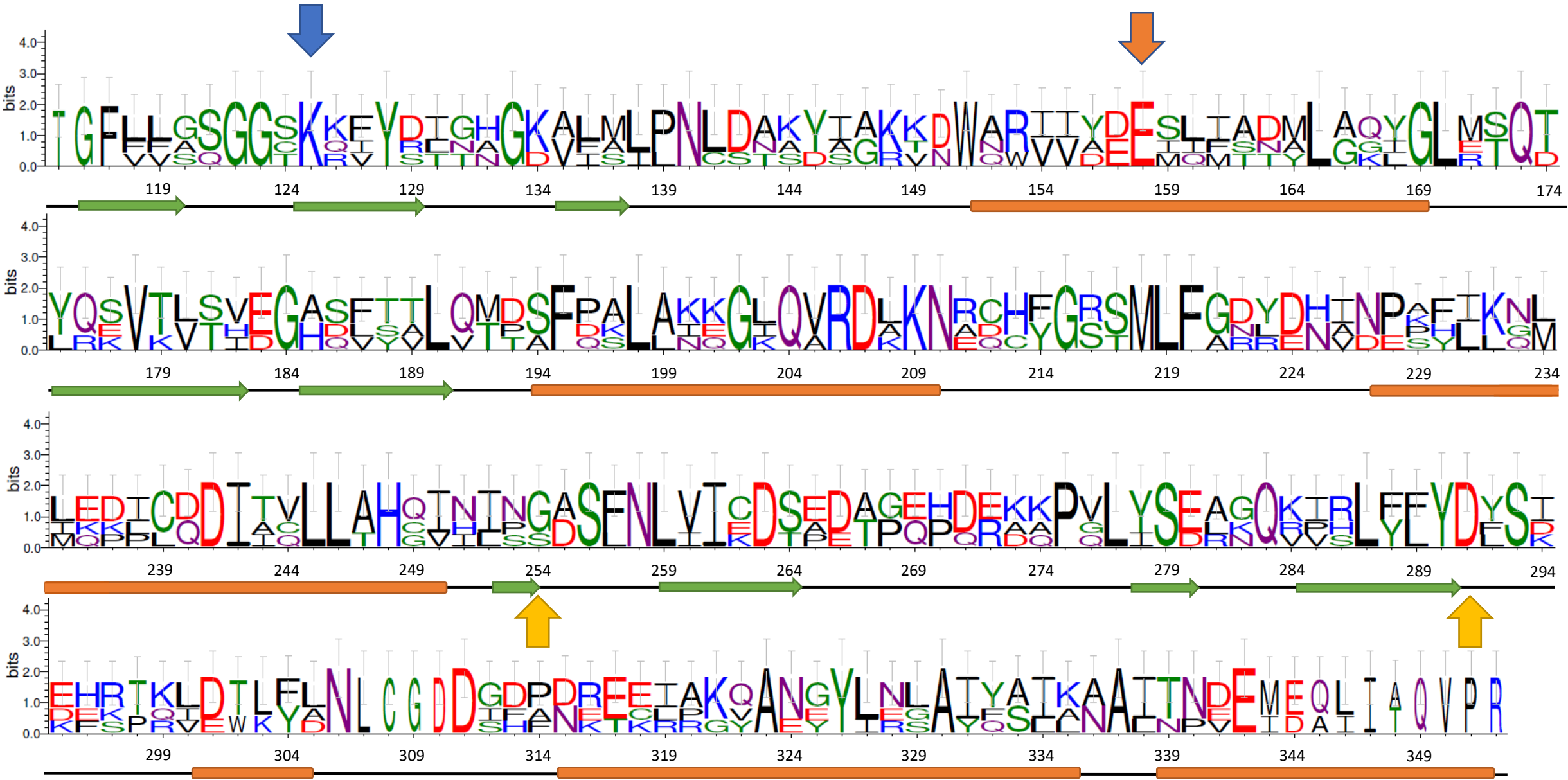

Supplement: Supplementary file 8 — Supplementary Information 8. [file 41598_2022_26109_MOESM8_ESM.pdf]

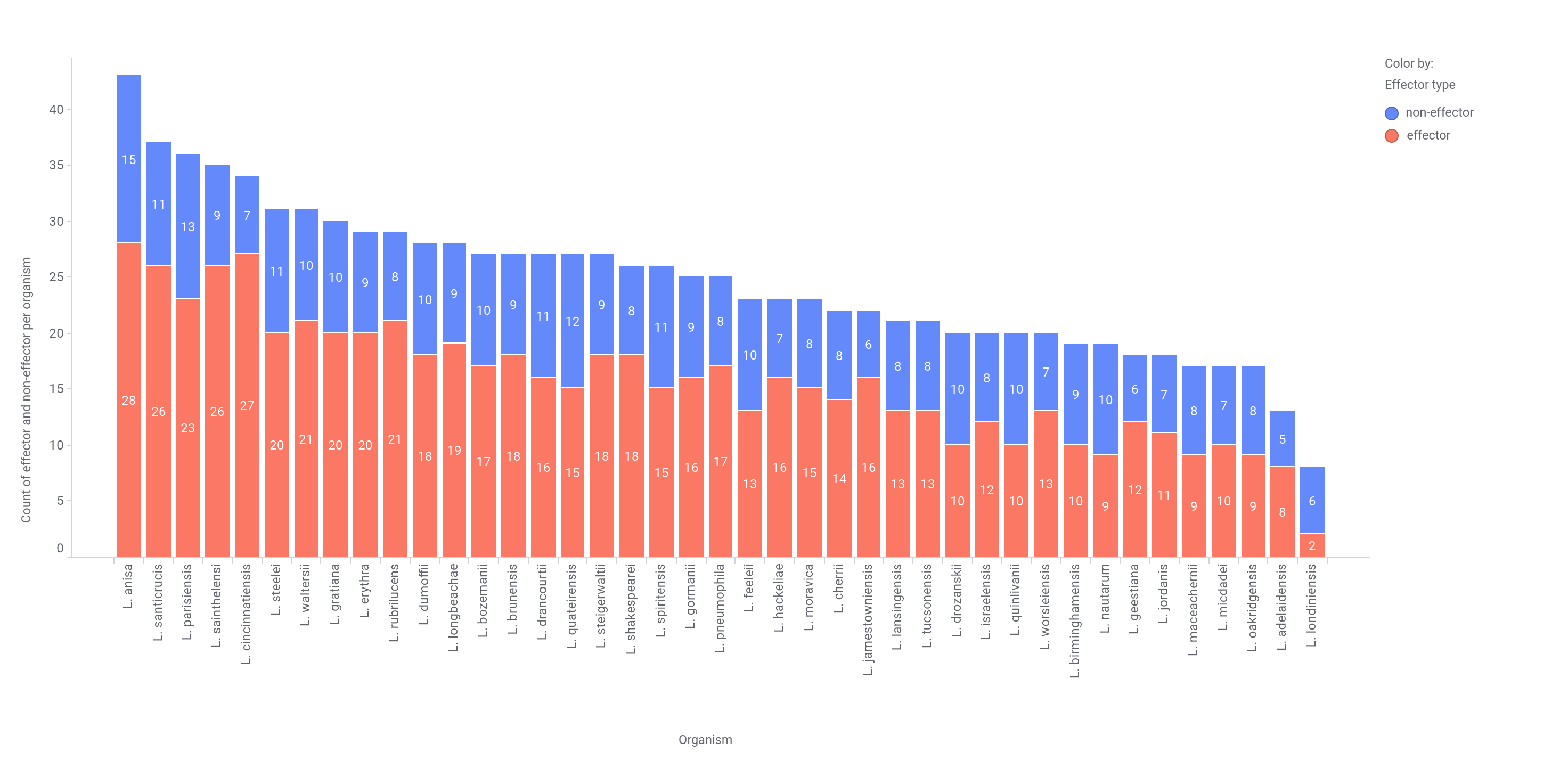

Supplement: Supplementary file 12 — Supplementary Information 12. [file 41598_2022_26109_MOESM12_ESM.tif]

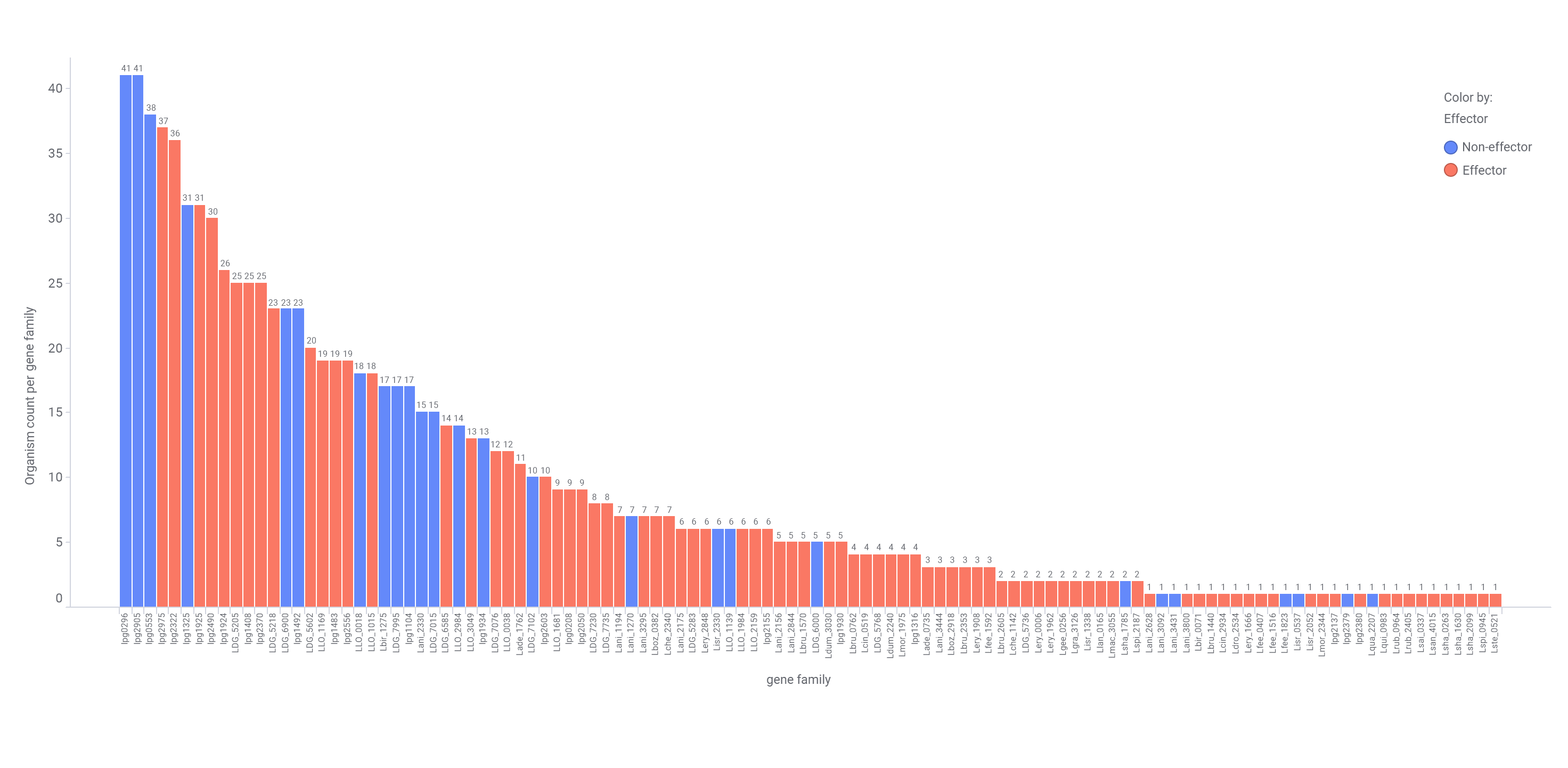

Supplement: Supplementary file 13 — Supplementary Information 13. [file 41598_2022_26109_MOESM13_ESM.tif]

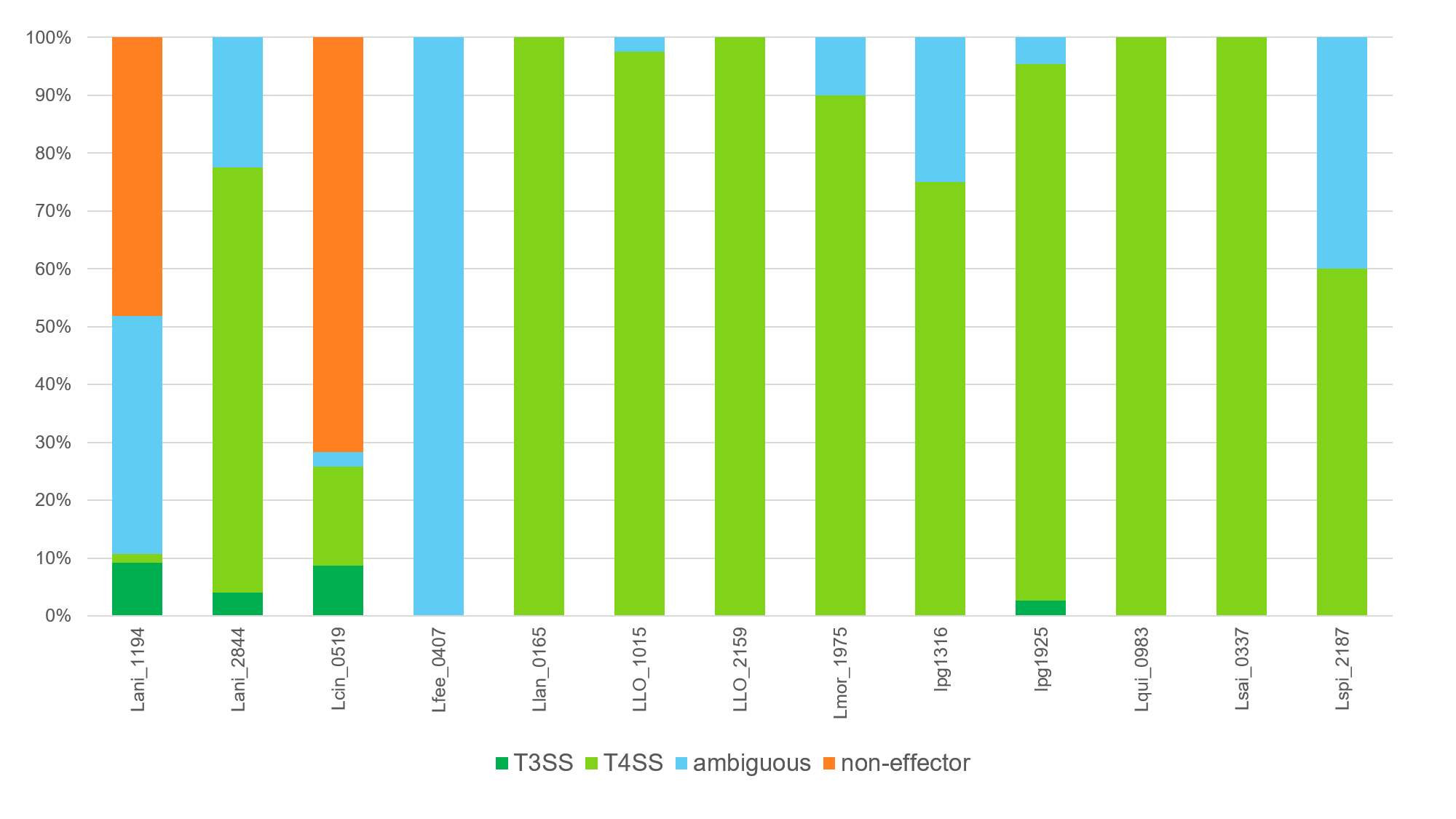

Supplement: Supplementary file 14 — Supplementary Information 14. [file 41598_2022_26109_MOESM14_ESM.png]

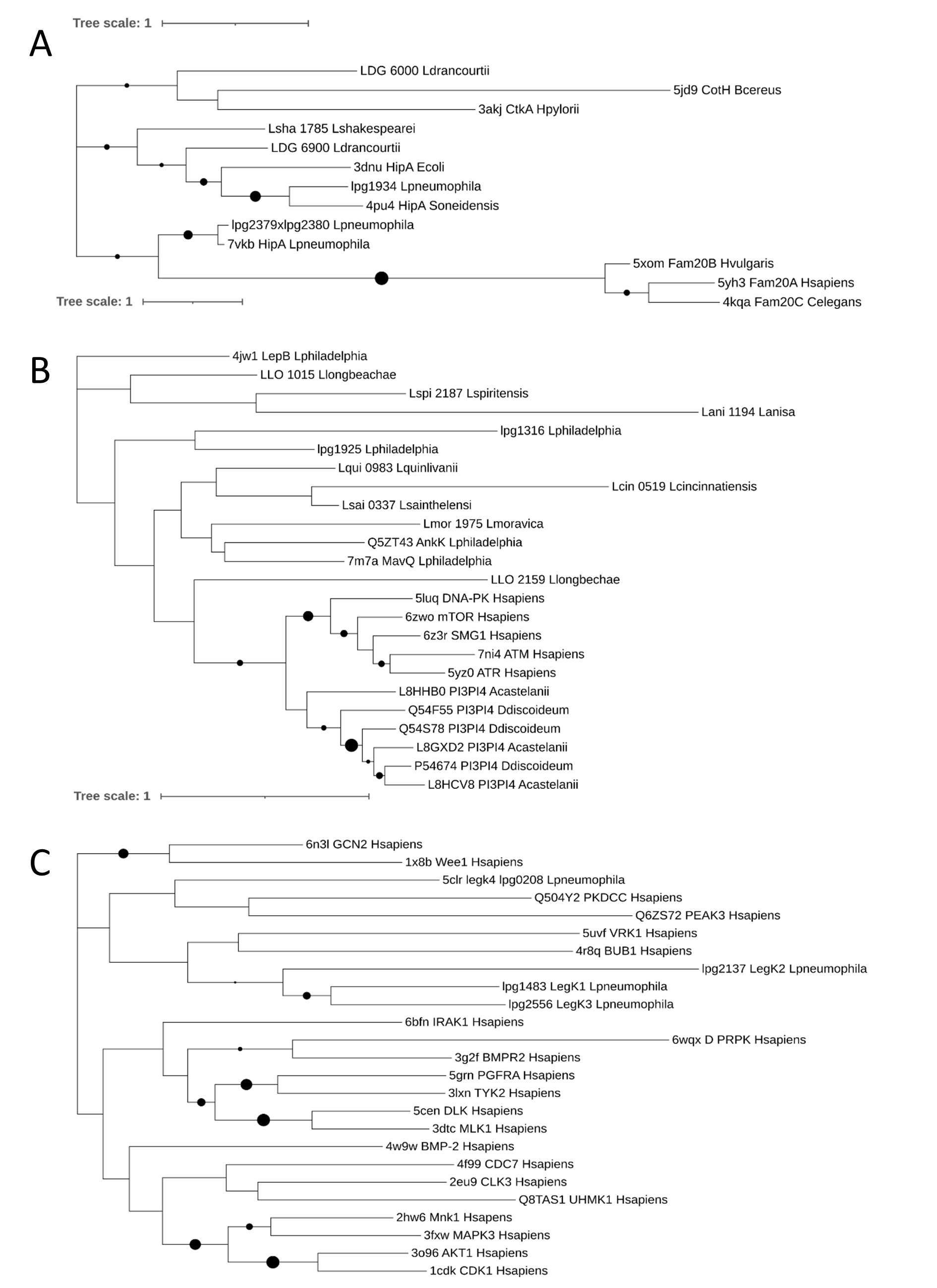

Supplement: Supplementary file 15 — Supplementary Information 15. [file 41598_2022_26109_MOESM15_ESM.tif]
