# Supplementary material for: Pan-kinome of Legionella expanded by a bioinformatics survey
Source: Sci Rep. 2022 Dec 16;12:21782. doi: 10.1038/s41598-022-26109-x (PMC9758233; doi:10.1038/s41598-022-26109-x)
Supplement: Supplementary file 16 — Supplementary Legends. [file 41598_2022_26109_MOESM16_ESM.docx]

**Pan-kinome of Legionella expanded by a bioinformatics survey**

**Supplementary Materials**

S1 – Table of CD-search and Pfamscan results

S2 – Table of FFAS, HHsearch and Phyre2 results

S3 – Table of structure modeling and structure searches

S4 – Table of effector prediction (*Legionella* pankinome, novel kinase families and HipA proteins)

S5 – Table of Lani_1194 and Lcin_0519 genomic neighborhood domains prediction

S6 – Table of Lani_1194 and Lcin_0519 neighborhood conservation analysis

S7 – Table of Lani_1194 and Lcin_0519 gene neighborhoods effector prediction

S8 – Suppl. Figure – Sequence logos of novel kinases

S9 – Data – 3D structure models of novel kinases

S10 – Data – Clans file Known_and_novel_kinase_families

S11 – Data – Clans file Legionella_and_host_kinases

S12 – Suppl. Figure. Effector and non-effector kinase distribution per *Legionella* species

S13 – Suppl. Figure. Kinase gene distribution in *Legionella*

S14 – Suppl. Figure. The percentage of predicted secretion system type III and IV substrates among the novel kinase-like families

S15 – Suppl. Figure. Phylogenetic trees for three Protein Kinase-Like groups from *Legionella* and their selected eukaryotic counterparts.

**Supplementary Figure Legends**

Suppl. Figure 8. Sequence logos for the 13 novel kinase-like families. Secondary structures represented as green arrows (beta strands) and brown shapes (alpha helices) Arrows denote predicted active site residues: equivalents of PKA D166 and D184 (yellow), K72 (blue) and E91 (brown).

Suppl. Figure 12. Effector and non-effector kinase distribution per *Legionella* species. Effector kinases shown in red, non-effectors – in blue. The Y-axis shows the number of kinase families per species.

Suppl. Figure 13. Protein kinase-like genes family frequency. The Y-axis shows organism count per representative gene (range 1–41). The X-axis shows gene family labels.

Suppl. Figure 14. The percentage of predicted secretion system type III and IV substrates among the novel kinase-like families.

Suppl. Figure 15. Phylogenetic trees (Maximum Likelihood with bootstrap) for selected families of Legionella kinases and some eukaryotic counterparts. A) HipA-like kinases, B) PI3-PI4 kinase-like proteins, C) Eukaryotic-like kinases (Legionella) and Ser/Thr kinases (eukaryotes). The underlying sequence alignments done using the structure alignment program mTM-align. Black dots indicate branches with bootstrap value > 50%.
